# GM1 Oligosaccharide Ameliorates Rett Syndrome Phenotypes In Vitro and In Vivo via Trk Receptor Activation

**DOI:** 10.3390/ijms252111555

**Published:** 2024-10-28

**Authors:** Maria Fazzari, Giulia Lunghi, Emma Veronica Carsana, Manuela Valsecchi, Eleonora Spiombi, Martina Breccia, Silvia Rosanna Casati, Silvia Pedretti, Nico Mitro, Laura Mauri, Maria Grazia Ciampa, Sandro Sonnino, Nicoletta Landsberger, Angelisa Frasca, Elena Chiricozzi

**Affiliations:** 1 Department of Medical Biotechnology and Translational Medicine, Università degli Studi di Milano, 20054 Segrate, Italy; giulia.lunghi@unimi.it (G.L.); emma.carsana@unimi.it (E.V.C.); manuela.valsecchi@unimi.it (M.V.); eleonora.spiombi@gmail.com (E.S.); martina.breccia@unimi.it (M.B.); silvia.casati@unimi.it (S.R.C.); laura.mauri@unimi.it (L.M.); maria.ciampa@unimi.it (M.G.C.); sandro.sonnino@unimi.it (S.S.); nicoletta.landsberger@unimi.it (N.L.); angelisa.frasca@unimi.it (A.F.); 2 Department of Pharmacological and Biomolecular Sciences “Rodolfo Paoletti”, Università degli Studi di Milano, 20133 Milan, Italy; silvia.pedretti@unimi.it (S.P.); nico.mitro@unimi.it (N.M.); 3 Department of Experimental Oncology, IEO, European Institute of Oncology IRCCS, 20139 Milan, Italy

**Keywords:** Rett syndrome, neurotrophins’ signaling, mitochondrial oxidative stress, GM1 ganglioside, GM1 oligosaccharide

## Abstract

Rett syndrome (RTT) is a severe neurodevelopmental disorder primarily caused by mutations in the methyl-CpG binding protein 2 (*MECP2*) gene. Despite advancements in research, no cure exists due to an incomplete understanding of the molecular effects of MeCP2 deficiency. Previous studies have identified impaired tropomyosin receptor kinase (Trk) neurotrophin (NTP) signaling and mitochondrial redox imbalances as key drivers of the pathology. Moreover, altered glycosphingolipid metabolism has been reported in RTT. GM1 ganglioside is a known regulator of the nervous system, and growing evidence indicates its importance in maintaining neuronal homeostasis via its oligosaccharide chain, coded as GM1-OS. GM1-OS directly interacts with the Trk receptors on the cell surface, triggering neurotrophic and neuroprotective pathways in neurons. In this study, we demonstrate that GM1-OS ameliorates RTT deficits in the *Mecp2*-null model. GM1-OS restored synaptogenesis and reduced mitochondrial oxidative stress of *Mecp2*-knock-out (ko) cortical neurons. When administered in vivo, GM1-OS mitigated RTT-like symptoms. Our findings indicate that GM1-OS effects were mediated by Trk receptor activation on the neuron’s plasma membrane. Overall, our results highlight GM1-OS as a promising candidate for RTT treatment.

## 1. Introduction

RTT syndrome (OMIM 312750) is a severe X-linked neurological disorder in children characterized by cognitive and motor impairments, stereotypic hand movements, and epilepsy. Affecting approximately 1 in 10,000 live-born girls, RTT is one of the leading causes of severe intellectual disability in females [1].

About 90% of RTT cases are caused by mutations in the *MECP2* gene, which encodes a multifunctional protein involved in gene expression regulation [2,3]. Animal models with *Mecp2* mutations, such as hemizygous *Mecp2*-ko or *Mecp2*-null mice, accurately replicate RTT symptoms and are widely used in RTT research. *Mecp2*-ko mice appear phenotypically normal until 3–4 weeks of age, when they develop RTT-like phenotypes such as hind limb clasping, stiff and uncoordinated gait, hypotonia, reduced movements, breathing irregularities, seizures, and premature death [4,5,6]. At cellular level, Mecp2 deficiency results in profound morphological alterations in neurons, including decreased soma dimension and dendritic arborization, dendritic spine dysgenesis, reduction of synapses’ number, and impaired synaptic plasticity [7,8,9,10,11].

One of the most studied molecular targets of MeCP2 is the brain-derived neurotrophic factor (BDNF) [12], a NTP essential for neuronal development, maturation, and synaptic plasticity [13]. BDNF specifically binds to and activates TrkB on the plasma membrane (PM), initiating downstream signaling pathways [13]. RTT brain samples revealed reduced BDNF protein and mRNA levels [12,14,15,16,17,18] and BDNF overexpression in *Mecp2*-null mice led to symptomatic improvements [14]. Reduced Nerve Growth Factor (NGF) and TrkA expression have also been found in the frontal cortex of RTT patients, further implicating the involvement of NTP signaling in RTT pathophysiology [19]. Accordingly, the therapeutic potential of NGF for RTT has been recently proposed [20,21].

In addition to its role in neuronal plasticity, BDNF has been shown to alter mitochondrial oxidative efficiency by improving respiratory control index via mitochondrial complex I and the Mitogen-activated protein kinase (MEK)-kinase cascade [22,23]. This suggests a link between impaired NTP signaling and mitochondrial dysfunction in RTT [24,25]. Indeed, RTT brains and neurons exhibit heightened oxidation, excess reactive oxygen species (ROS) production, compromised ROS scavenger systems, reduced levels of adenosine triphosphate (ATP), and defects in mitochondrial respiration and cristae integrity [21,26,27,28,29].

As a transmembrane protein, the NTP receptors activity is influenced by their surrounding lipid microenvironment, and GM1 ganglioside plays a crucial role in regulating their function [30,31]. GM1 is essential for the central nervous system homeostasis, and altered GM1 levels are associated with neurodegenerative diseases such as Alzheimer’s, Parkinson’s, and Huntington’s. Exogenous GM1 administration has been shown to alleviate pathological signs in several in vitro and in vivo neurodegenerative models and in patients [30,32,33,34]. Interestingly, in *Mecp2*-null mice [35] and RTT patients [36,37], reduced levels of GD1a ganglioside, the catabolic precursor of GM1, have been observed.

GM1 consists of a lipid moiety, the ceramide, embedded in the outer leaflet of the PM, and a hydrophilic oligosaccharide chain, protruding in the extracellular space [30]. Recent studies have identified the GM1-OS as the bioactive component responsible for neuroprotective and neurotrophic effects by interacting with the cell surface’s NTP receptors like TrkA [38,39,40,41,42,43,44,45,46,47,48]. This interaction regulates key neuronal processes, including cell migration, clustering, and adhesion [38,42], mitochondrial function [44], oxidative stress response [40,46,48], excitotoxicity [48], and calcium signaling [43].

In the present work, we investigated the therapeutic potential of GM1-OS in the *Mecp2*-ko model of RTT. In vitro, GM1-OS restored synaptogenesis and countered mitochondrial oxidative stress. In vivo, GM1-OS administration improved RTT-like symptoms and motor deficits. Our findings indicate that GM1-OS mitigated RTT phenotypes by activating Trk receptor activity on PM.

## 2. Results

### 2.1. GM1-OS Recovers Synaptic Defects in Mecp2-ko Neurons

It has been proposed that MeCP2 activity is crucial for the maintenance of functional mature neurons [2]. Accordingly, *Mecp2*-ko neurons exhibit a consistent reduction of dendritic arborization and network and alterations at dendritic spines resulting in defects in synaptic transmission and plasticity [7,8,9,10,11].

We have previously demonstrated that GM1-OS administration to primary neurons is able to accelerate the maturation process, triggering the formation of neuronal clusters by increasing the expression of constituents of neuronal processes and synaptic density, as well as the expression of complex gangliosides [42].

**Figure 1:**
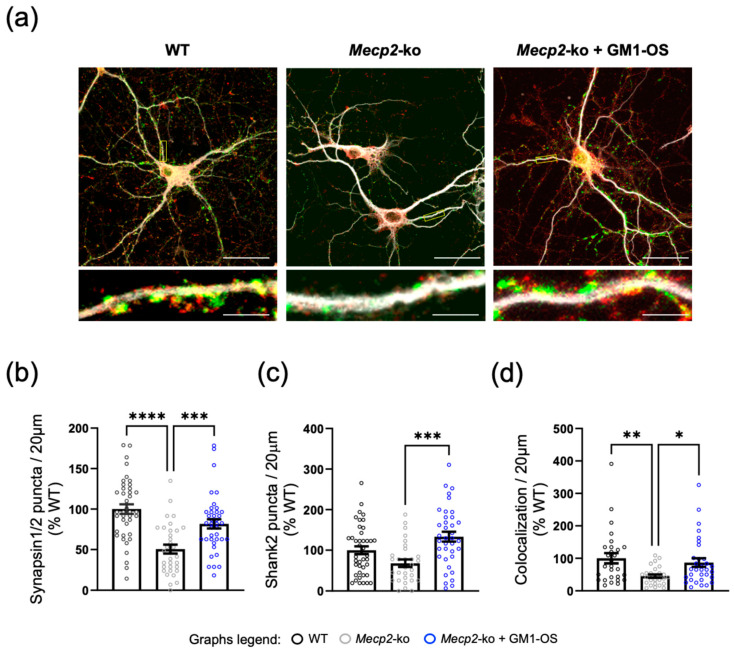
GM1-OS activity at synaptic compartment in RTT neurons. (**a**) Representative images of WT and *Mecp2*-null neurons immunostained for microtubule-associated protein 2 (MAP2, white), Syn (green), and Shank2 (red). Images have been equally adjusted for brightness and contrast. GM1-OS or vehicle were added to the culture medium at DIV0, and neurons were cultured for 14 days. For each condition, the overlay of the signals is displayed (63×magnification, scale bar 30 μm and 5 μm in the enlarged image). (**b**–**d**) Histograms indicate mean ± SEM of number of Syn (mean ± SEM: WT = 100 ± 5.95; *Mecp2*-ko = 50.62 ± 5.46; *Mecp2*-ko + GM1-OS = 81.72 ± 5.70) and Shank2 (mean ± SEM: WT = 100 ± 9.66; *Mecp2*-ko = 68.31 ± 8.98; *Mecp2*-ko + GM1-OS = 133.3 ± 12.04) puncta in 20 μm of proximal dendrites (**b**,**c**) and of colocalized puncta (mean ± SEM: WT = 100 ± 15.62; *Mecp2*-ko = 45.47 ± 5.34; *Mecp2-ko* + GM1-OS = 86.86 ± 12.88) (**d**) expressed as percentage respect to WT controls (set at 100%). Neurons derived from a pool of at least three different biological replicates for each genotype; at least 30 neurons for each experimental condition were analyzed (Syn: *p* < 0.0001 WT vs. *Mecp2*-ko; *p* = 0.0586 WT vs. *Mecp2*-ko + GM1-OS; *p* = 0.0009 *Mecp2*-ko vs. *Mecp2*-ko + GM1-OS; Shank2: *p* = 0.096 WT vs. *Mecp2*-ko; *p* = 0.0558 WT vs. *Mecp2*-ko + GM1-OS; *p* = 0.0002 *Mecp2*-ko vs. *Mecp2*-ko + GM1-OS; Colocalization: *p* = 0.0085 WT vs. *Mecp2*-ko; *p* = 0.7246 WT vs. *Mecp2*-ko + GM1-OS; *p* = 0.0496 *Mecp2*-ko vs. *Mecp2*-ko + GM1-OS; **** *p* < 0.0001; *** *p* < 0.001, ** *p* < 0.01, * *p* < 0.05 by one-way ANOVA followed by Tukey post-hoc test).

Thus, we aimed to verify whether GM1-OS can exert beneficial effects on *Mecp2*-deficient neurons maturation by using in vitro cultures of primary cortical neurons. As a readout of neuronal maturation, we analyzed synaptogenesis via immunostaining of synaptic puncta at 14 days in vitro (DIV14) with antibodies recognizing pre-synaptic (Synapsin 1/2, Syn) and post-synaptic (SH3 and multiple ankyrin repeat domains protein 2, Shank2) markers (Figure 1a). This time-point was selected as wild-type (WT) cortical neurons display fully functional synapses, while in *Mecp2*-ko neurons defects in synaptogenesis were demonstrated [49,50,51]. By quantifying confocal images, we confirmed the reduction of Syn (mean ± standard error of the mean, SEM: WT = 100 ± 5.95, *Mecp2*-ko = 50.62 ± 5.46, *p* < 0.0001 WT vs. *Mecp2*-ko) and Shank2 (mean ± SEM: WT = 100 ± 9.66, *Mecp2*-ko = 68.31 ± 8.98, *p* = 0.096 WT vs. *Mecp2*-ko) puncta in *Mecp2*-null neurons with respect to WT neurons, as already described in literature [49,50] (Figure 1b,c). Conversely, GM1-OS administration for 14 days was able to significantly increase their levels (mean ± SEM of Syn of *Mecp2*-ko + GM1-OS = 81.72 ± 5.70, *p* = 0.0009 *Mecp2*-ko vs. *Mecp2*-ko + GM1-OS; mean ± SEM of Shank2 of *Mecp2*-ko + GM1-OS = 133.3 ± 12.04, *p* = 0.0002 *Mecp2*-ko vs. *Mecp2*-ko + GM1-OS; Figure 1). Moreover, analyzing the colocalization between Syn and Shank2 puncta, we assessed that *Mecp2*-null neurons displayed a significant recovery of the number of functional synapses upon GM1-OS treatment, restoring the physiological condition (mean ± SEM: WT = 100 ± 15.62; *Mecp2*-ko = 45.47 ± 5.34; *Mecp2-ko* + GM1-OS = 86.86 ± 12.88, *p* = 0.0496 *Mecp2*-ko vs. *Mecp2*-ko + GM1-OS; Figure 1d).

These results indicate that GM1-OS can support RTT neuron maturation in vitro by favoring synaptogenesis.

### 2.2. GM1-OS Reduces the Excess of Mitochondrial Superoxide (O_2_^•−^) Due to Mecp2 Loss

Redox imbalance and oxidative stress have recently emerged as key features in RTT pathology. Accordingly, mitochondria, the main cellular site for ROS production, result impaired in RTT [21,25,26,27,28,29].

On the other hand, GM1-OS has been shown to enhance mitochondrial function and counteract oxidative stress in different experimental models [40,44,46,48].

Based on these premises, we analyzed the expression of mitochondria-localized proteins in WT and *Mecp2*-null neurons cultured in the absence or presence of GM1-OS for 7 days; this time-point was selected since cellular impairments are already evident in *Mecp2*-null cortical neurons [49,50,51].

**Figure 2:**
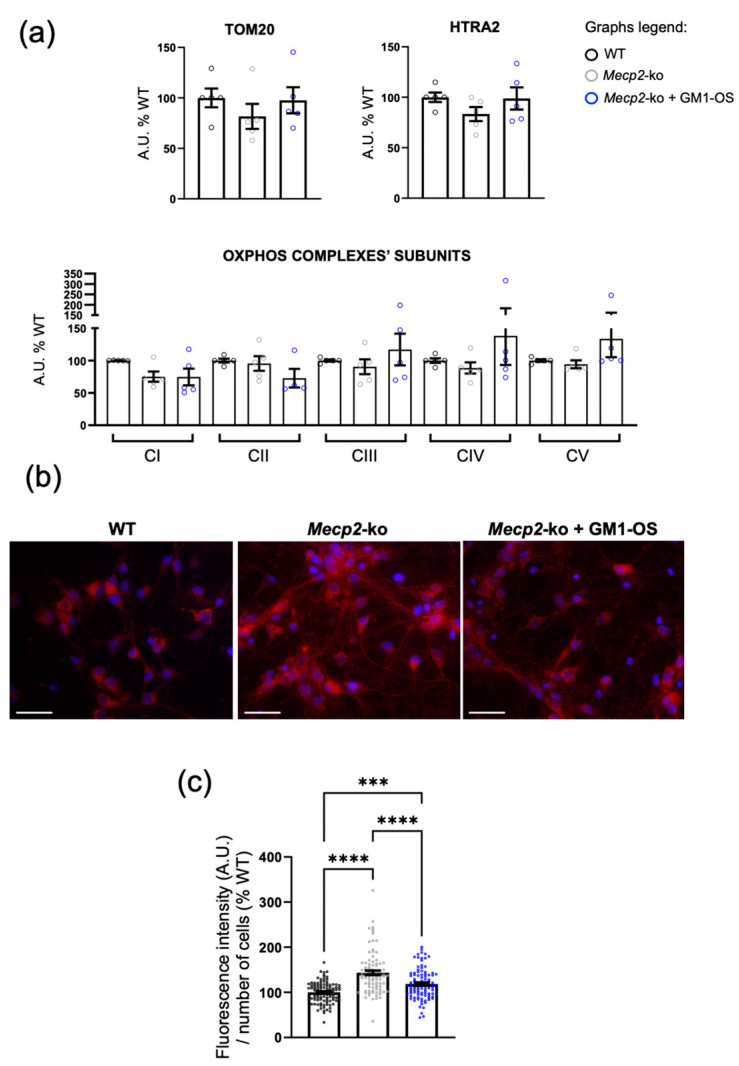
GM1-OS function at mitochondria in RTT neurons. (**a**) Expression level of mitochondrial proteins in WT and *Mecp2*-null neurons (DIV7) treated with vehicle or 50 μM GM1-OS for 7 days and evaluated by western blotting (WB). Histograms depict the mean ± SEM of translocase of outer mitochondrial membrane 20 (TOM20, top, on the left, mean ± SEM: WT = 100 ± 9.29; *Mecp2*-ko = 81.67 ± 12.34; *Mecp2*-ko + GM1-OS = 97.61 ± 12.92), high-temperature requirement protein A2 (HTRA2, top, on the right, mean ± SEM: WT = 100 ± 4.74; *Mecp2*-ko = 83.4 ± 6,96; *Mecp2*-ko + GM1-OS = 98.8 ± 11), and oxidative phosphorylation (Oxphos) complexes (bottom, mean ± SEM of CI: WT = 100 ± 0.18; *Mecp2*-ko = 75.2 ± 7.92; *Mecp2*-ko + GM1-OS = 74.63 ± 12.93; mean ± SEM of CII: WT = 100 ± 2.89; *Mecp2*-ko = 95.5 ± 11.25; *Mecp2*-ko + GM1-OS = 72.84 ± 14,4; mean ± SEM of CIII: WT = 100 ± 1.66; *Mecp2*-ko = 90.52 ± 11.5; *Mecp2*-ko + GM1-OS = 117.1 ± 24.42; mean ± SEM of CIV: WT = 100 ± 3.44; *Mecp2*-ko = 88.6 ± 8.72; *Mecp2*-ko + GM1-OS = 138.2 ± 45.02; mean ± SEM of CV: WT = 100 ± 1.93; *Mecp2*-ko = 94.3 ± 6.11; *Mecp2*-ko + GM1-OS = 133.5 ± 28.3) expressed as percentage respect to WT controls (set at 100%). Data were obtained by dividing the signal intensity of mitochondrial proteins by the ones of heat shock protein 90 (HSP90) or calnexin, the latter used as loading controls (see Supplementary Figure S1 for WB representative images). Neuron lysates derived from a pool of at least three different biological replicates obtained from three independent experiments (TOM20: *p* = 0.5236 WT vs. *Mecp2*-ko; *p* = 0.9884 WT vs. *Mecp2*-ko + GM1-OS; *p* = 0.6091 *Mecp2*-ko vs. *Mecp2*-ko + GM1-OS; HTRA2: *p* = 0.3394 WT vs. *Mecp2*-ko; *p* = 0.9937 WT vs. *Mecp2*-ko + GM1-OS; *p* = 0.3903 *Mecp2*-ko vs. *Mecp2*-ko + GM1-OS; CI: *p* = 0.1537 WT vs. *Mecp2*-ko; *p* = 0.1429 WT vs. *Mecp2*-ko + GM1-OS; *p* = 0.9989 *Mecp2*-ko vs. *Mecp2*-ko + GM1-OS; CII: *p* = 0.9425 WT vs. *Mecp2*-ko; *p* = 0.1947 WT vs. *Mecp2*-ko + GM1-OS; *p* = 0.3046 *Mecp2*-ko vs. *Mecp2*-ko + GM1-OS; CIII: *p* = 0.9042 WT vs. *Mecp2*-ko; *p* = 0.7256 WT vs. *Mecp2*-ko + GM1-OS; *p* = 0.4739 *Mecp2*-ko vs. *Mecp2*-ko + GM1-OS; CIV: *p* = 0.9505 WT vs. *Mecp2*-ko; *p* = 0.5797 WT vs. *Mecp2*-ko + GM1-OS; *p* = 0.4100 *Mecp2*-ko vs. *Mecp2*-ko + GM1-OS; CV: *p* = 0.9683 WT vs. *Mecp2*-ko; *p* = 0.3648 WT vs. *Mecp2*-ko + GM1-OS; *p* = 0.2612 *Mecp2*-ko vs. *Mecp2*-ko + GM1-OS by one-way ANOVA followed by Tukey post-hoc test. (**b**) Representative fluorescence images of cortical neurons (DIV7) cultured in presence of 50 μM GM1-OS or vehicle for 7 days and stained with MitoSOX™ Red reagent. Images have been equally adjusted for brightness and contrast. For each condition, the overlay of MitoSOX in red and Nuclei in blue (scale bar 30 μm, 40× magnification) is displayed. (**c**) Quantification of the MitoSOX Red signal over nuclei number (mean ± SEM: WT = 100 ± 2.35; *Mecp2*-ko = 143.5 ± 4.83; *Mecp2*-ko + GM1-OS = 118.6 ± 3.4). All values are expressed as mean ± SEM of values from three independent experiments in which pools of at least three different biological replicates per genotype were employed (*p* < 0.0001 WT vs. *Mecp2*-ko; *p* = 0.0007 WT vs. *Mecp2*-ko + GM1-OS; *p* < 0.0001 *Mecp2*-ko vs. *Mecp2*-ko + GM1-OS; **** *p* < 0.0001; *** *p* < 0.001 by one-way ANOVA followed by Tukey post-hoc test).

As shown in Figure 2a and in Supplementary Figure S1, no significant deregulation of analyzed mitochondrial proteins can be observed in *Mecp2*-null neurons at DIV7, and only a tendency of increased expression of complex IV (cytochrome c oxidase, mean ± SEM: WT = 100 ± 3.44; *Mecp2*-ko = 88.6 ± 8.72; *Mecp2*-ko + GM1-OS = 138.2 ± 45.02; *p* = 0.4100 *Mecp2*-ko vs. *Mecp2*-ko + GM1-OS.) and complex V (ATP-synthase, mean ± SEM: WT = 100 ± 1.93; *Mecp2*-ko = 94.3 ± 6.11; *Mecp2*-ko + GM1-OS = 133.5 ± 28.3; *p* = 0.2612 *Mecp2*-ko vs. *Mecp2*-ko + GM1-OS) subunits is evident in GM1-OS treated samples.

We thus investigated whether oxidative stress occurs in our experimental conditions. To this purpose, the level of mitochondrial O_2_^•−^ was evaluated by staining neurons with the specific MitoSOX^TM^ Red dye at the end of the 7-day treatment (Figure 2b). In accordance with literature [24,26,27,29], the analysis revealed that Mecp2 deficiency determines an increase of mitochondrial oxidative stress (mean ± SEM: WT = 100 ± 2.35; *Mecp2*-ko = 143.5 ± 4.83; *p* < 0.0001 WT vs. *Mecp2*-ko). Interestingly, GM1-OS treatment significantly lowered O_2_^•−^ overabundance in *Mecp2*-null neurons (mean ± SEM: *Mecp2*-ko + GM1-OS = 118.6 ± 3.4; *p* < 0.0001 *Mecp2*-ko vs. *Mecp2*-ko + GM1-OS; Figure 2c).

These outcomes suggest that GM1-OS can support RTT neurons’ homeostasis by counteracting mitochondrial oxidative stress.

### 2.3. GM1-OS Ameliorates RTT-like Symptoms in Mecp2-ko Mice

**Figure 3:**
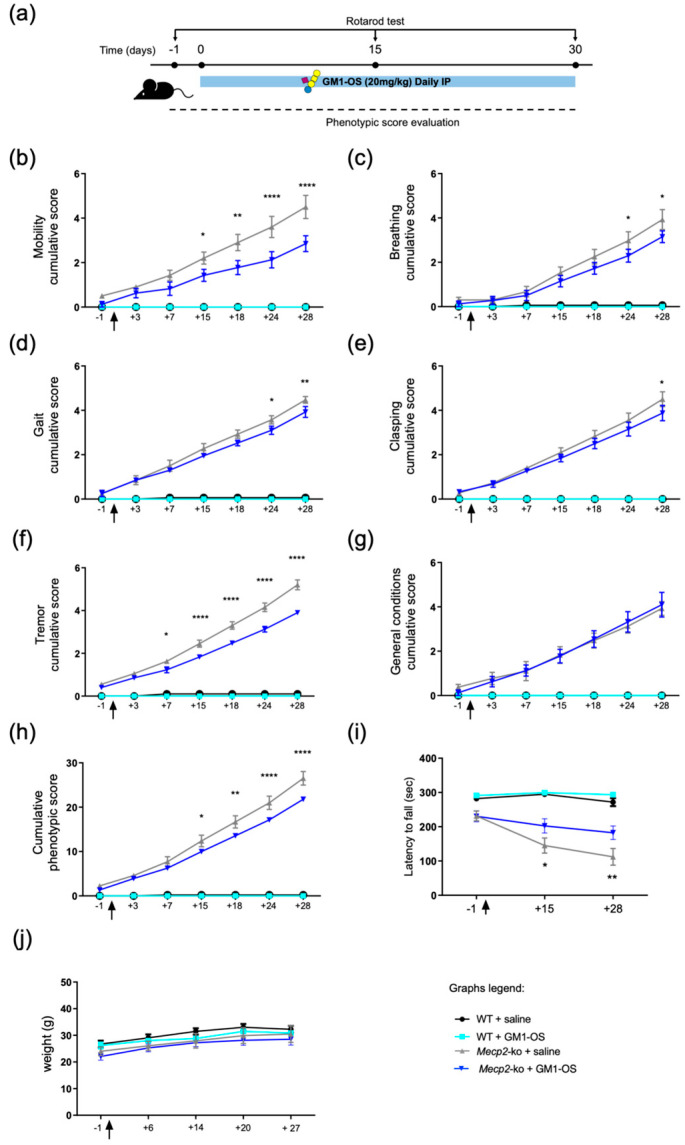
GM1-OS mitigation of RTT-like features in vivo. (**a**) GM1-OS (20 mg/kg) or saline was injected in WT and *Mecp2*-ko (P35–37) for 4 weeks; behavioral tests were conducted before, along, and at the end of the treatment. (**b**–**h**) The graphs indicate the mean ± SEM of mobility (**b**), breathing (**c**), gait (**d**), clasping (**e**), tremor (**f**), general conditions (**g**), cumulative phenotypic score (**h**), accelerated rotarod (**i**), and weight (**j**). Black arrow indicates the start of GM1-OS/vehicle administration. Numbers on x axis indicate the days before treatment if preceded by minus sign or during the treatment if preceded by plus sign. Asterisks denote a significant difference between *Mecp2*-ko receiving GM1-OS and vehicle injection evaluated by two-way ANOVA followed by Tukey post-hoc test (**** *p* < 0.0001; ** *p* < 0.01; * *p* < 0.05; see Supplementary Tables for exact *p*-values and mean ± SEM of each condition and time-point). WT + saline, *n* = 5; WT + GM1-OS, *n* = 5; *Mecp2*-ko + saline, *n* = 4; *Mecp2*-ko + GM1-OS, *n* = 4 (graphs **b**–**h**). WT + saline, *n* = 11; WT + GM1-OS, *n* = 6; *Mecp2*-ko + saline, *n* = 10; *Mecp2*-ko + GM1-OS, *n* = 9 (graph **i**). WT + saline, *n* = 6; WT + GM1-OS, *n* = 6; *Mecp2*-ko + saline, *n* = 6; *Mecp2*-ko + GM1-OS, *n* = 5 (graph **j**).

To assess the potential therapeutic effect in vivo, GM1-OS (20 mg/kg in physiological saline solution) or saline solution (control) was, every day, intraperitoneally (IP) injected in WT or *Mecp2*-null mice (postnatal days 35–37, P35–37) for 4 weeks (Figure 3a).

WT and *Mecp2*-null animals were assigned to different groups (saline- or GM1-OS-treated) by randomization, and all the analyses were conducted by a researcher blind to treatment.

To measure the starting basal conditions and to monitor mice during the treatment, RTT-like symptoms were evaluated according to the well-defined Guy’s scoring system [6,52], and motor performance was tested before and along the treatment. The graphs depicted in Figure 3b–h clearly highlight the presence of RTT-like symptoms in *Mecp2*-ko mice compared with WT ones, as indicated by the score assigned to mobility, breathing, gait, clasping, tremor, and general condition and summarized in the cumulative phenotypic score (see Supplementary Tables S1–S7 for mean, SEM, and *p*-values for each parameter and time-point). Importantly, Figure 3h indicates that GM1-OS significantly ameliorated RTT-like symptoms starting from roughly 15 days of treatment (mean ± SEM: *Mecp2*-ko + saline = 12.37 ± 1.32; *Mecp2*-ko + GM1-OS = 9.97 ± 0.56; *p* = 0.0281 *Mecp2*-ko + saline vs. *Mecp2*-ko + GM1-OS).

To better understand GM1-OS effectiveness, motor coordination and balance were tested via an accelerated rotarod test [53]. As shown in Figure 3i, GM1-OS was able to counteract the worsening of the motor defects (see Supplementary Table S8 for mean, SEM, and *p*-values for each time-point). Indeed, the *Mecp2*-null control group and GM1-OS receiving group demonstrated an identical performance on day −1 (mean ± SEM: *Mecp2*-ko + saline = 231.46 ± 14.32; *Mecp2*-ko + GM1-OS = 230.67 ± 16.22; *p* > 0.9999 *Mecp2*-ko + saline vs. *Mecp2*-ko + GM1-OS), while the two experimental groups were significantly different already after 2 weeks of treatment (mean ± SEM: *Mecp2*-ko + saline = 145.21 ± 21.94; *Mecp2*-ko + GM1-OS = 202.56 ± 20.61; *p* = 0.0461 *Mecp2*-ko + saline vs. *Mecp2*-ko + GM1-OS). Additionally, body weight was monitored over time, assessing no difference among the experimental groups (Figure 3j, Supplementary Table S9).

Altogether, these results indicate that GM1-OS can improve RTT-related symptoms in vivo by halting the progression of the disease.

### 2.4. GM1-OS Activates Trk Receptors

Previous work has demonstrated that GM1-OS interacts with cell surface receptors, prompting the differentiation and maturation in mouse neuroblastoma Neuro2a (N2a) cells and primary granule neurons [38,39,42].

**Figure 4:**
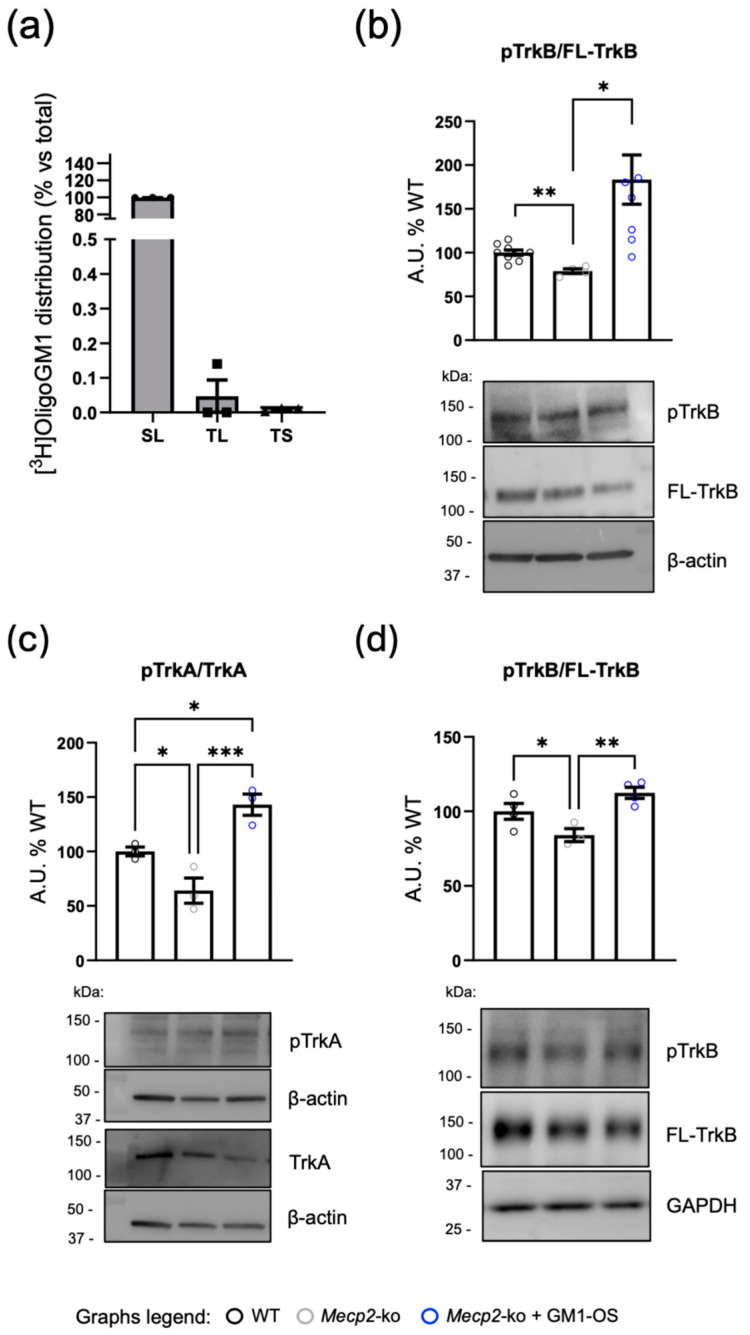
GM1-OS activation of Trk receptor at the PM in RTT models in vitro and in vivo. (**a**) Cortical neurons (DIV7) were incubated with 50 μM [^3^H]GM1-OS for 20 h. After pulse, the medium was withdrawn and collected. The cells were then washed with a medium supplemented with 10% fetal bovine serum (FBS) to gather SL fraction. The TL fraction was then obtained by washing the cells with a low concentrated trypsin solution. In order to collect the TS fraction, which represents the portion of the internalized GM1-OS, cells were finally lysed. Using liquid scintillation counting, the radioactivity associated with the medium and each fraction was ascertained. The graph displays the quantity of radioactivity that is still present after the medium has been removed. Data are expressed as mean ± SEM of total radioactivity percentage from three independent experiments. (**b**) Activation level of FL-TrkB receptor evaluated through WB by verifying the phosphorylation degree on lysates obtained from WT and *Mecp2*-null neurons (DIV7) treated with vehicle or 50 μM GM1-OS for 10 min. The graph depicts the degree of phosphorylation obtained by dividing the signal intensity of pTrkB by that of FL-TrkB. Data are expressed as mean ± SEM of the percentage respect to WT controls (mean ± SEM: WT = 100 ± 3.12; *Mecp2*-ko = 78.81 ± 2.8; *Mecp2*-ko + GM1-OS = 183.5 ± 28.05); values were obtained from three independent experiments in which pools of at least three different biological replicates per genotype were employed (*p* = 0.0014 WT vs. *Mecp2*-ko; *p* = 0.0572 WT vs. *Mecp2*-ko + GM1-OS; *p* = 0.0208 *Mecp2*-ko vs. *Mecp2*-ko + GM1-OS; ** *p* < 0.01, * *p* < 0.05 by Brown-Forsythe and Welch one-way ANOVA). (**c**,**d**) Activation level of TrkA and FL-TrkB receptor evaluated through WB by verifying the phosphorylation of TrkA (pTrkA, **c**) and of FL-TrkB (pTrkB, **d**) on lysates obtained from WT and *Mecp2*-null cortices of mice treated with saline or GM1-OS (20 mg/Kg) for 4 weeks (the last injection was performed 2 h before sacrifice). The graph shows the phosphorylation level that results from dividing the phosphorylation signal (normalized by β-actin or glyceraldehyde-3-phosphate dehydrogenase, GADPH) by the total TrkA or FL-TrkB intensity (normalized by β-actin or GADPH). Values are expressed as mean ± SEM of the percentage respect to WT controls (mean ± SEM of pTRKA/TRKA: WT = 100 ± 4.04; *Mecp2*-ko = 64 ± 11.53; *Mecp2*-ko + GM1-OS = 143 ± 9.71; mean ± SEM of pTRKB/FL-TRKB: WT = 100 ± 5.33; *Mecp2*-ko = 84.01 ± 4.4; *Mecp2*-ko + GM1-OS= 112.4 ± 3.8). Asterisks highlight a significant difference evaluated by one-way ANOVA followed by Fisher’s LSD post hoc test (pTrkA/TrkA: *p* = 0.0302 WT vs. *Mecp2*-ko; *p* = 0.0150 WT vs. *Mecp2*-ko + GM1-OS; *p* = 0.0008 *Mecp2*-ko vs. *Mecp2*-ko + GM1-OS; pTrkB/FL-TrkB: *p* = 0.0456 WT vs. *Mecp2*-ko; *p* = 0.083 WT vs. *Mecp2*-ko + GM1-OS; *p* = 0.003; *Mecp2*-ko vs. *Mecp2*-ko + GM1-OS; *** *p* < 0.001, ** *p* < 0.01, * *p* < 0.05). WT + saline, *n* = 4; *Mecp2*-ko + saline, *n* = 3; *Mecp2-ko* + GM1-OS, *n* = 3–4.

To elucidate whether the action of GM1-OS is mediated by interaction with PM proteins in the in vitro model used in this study, we investigated the fate of GM1-OS in WT cortical neurons (DIV7). Cells were cultured in the presence of isotopic tritium labeled [^3^H]GM1-OS for 20 h. At the end of treatment, the medium was collected, and neurons were subjected to sequential washes with culture medium containing 10% serum in order to collect the fraction of tritiated oligosaccharides weakly associated to the cell surface (serum labile fraction, SL). Then, cells were treated with low-concentrated trypsin to remove the possible fraction strongly bound to the cell surface (trypsin labile fraction, TL). Finally, cells were lysed to collect the fraction of the internalized [^3^H]GM1-OS corresponding to the trypsin stabile (TS) fraction. As shown in Figure 4a, we found that 99% GM1-OS was associated with the SL fraction, suggesting that the GM1-OS remained outside the cells; there it may weakly bind to cortical neurons PM and interact with PM receptors.

In particular, we proved that GM1-OS is able to directly interact, stabilize, and activate the NGF TrkA receptor, thus triggering a neurotrophic intracellular cascade [38,39,40,42,43].

In RTT, different studies documented the impairment of BDNF signaling, which is mediated by the PM activation of the TrkB receptor [12,14,15,16,17,18]. Additionally, a deregulation of the NGF-TrkA cascade has been suggested [19,20,21].

Thus, we decided to investigate the activation state of the full-length TrkB (FL-TrkB) receptor on DIV7 cortical neurons treated or not with 50 μM GM1-OS. By analyzing the autophosphorylation of the receptor by immunoblotting, a reduction of FL-TrkB activation is observed in *Mecp2*-null neurons compared with WT ones (mean ± SEM: WT = 100 ± 3.12; *Mecp2*-ko = 78.81 ± 2.8; *p* = 0.0014 WT vs. *Mecp2*-ko). Interestingly, GM1-OS induced a significant increase of FL-TrkB phosphorylation state (mean ± SEM: *Mecp2*-ko + GM1-OS = 183.5 ± 28.05; *p* = 0.0208 *Mecp2*-ko vs. *Mecp2*-ko + GM1-OS; Figure 4b).

This evidence was further confirmed by in vivo results. Indeed, a significant hypoactivation of both TrkA (mean ± SEM: WT = 100 ± 4.04; *Mecp2*-ko = 64 ± 11.53; *p* = 0.0302 WT vs. *Mecp2*-ko; Figure 4c) and FL-TrkB (mean ± SEM: WT = 100 ± 5.33; *Mecp2*-ko = 84.01 ± 4.4; *p* = 0.0456 WT vs. *Mecp2*-ko; Figure 4d) was detected in cerebral cortex lysates from *Mecp2*-null mice that was rescued upon a 4-week treatment with GM1-OS (mean ± SEM for pTrkA/TrkA: *Mecp2*-ko + GM1-OS = 143 ± 9.71; *p* = 0.0008 *Mecp2*-ko vs. *Mecp2*-ko + GM1-OS; Figure 4c; mean ± SEM for pTrkB/FL-TrkB: *Mecp2*-ko + GM1-OS = 112.4 ± 3.78; *p* = 0.003 *Mecp2*-ko vs. *Mecp2*-ko + GM1-OS; Figure 4d).

Altogether, this evidence suggests that the neurotrophic effect deriving from GM1-OS administration can be due to the modulation of the Trk cascade on PM.

## 3. Discussion

Recent studies from our group demonstrated that GM1 activities depend on its oligosaccharide portion, which alone promotes neurite outgrowth in N2a cells [38], accelerates neuronal maturation of primary cerebellar granule neurons [42], and protects against genetic- [41,48] or toxin-based neurodegeneration [40,45,46,48]. Specifically, we demonstrated that GM1-OS supports neuronal homeostasis by interacting on PM with the NGF TrkA receptor [38,39,42,43,47]. It is interesting to notice that, by losing the ceramide tail, GM1-OS acquires a significantly enhanced capability to cross the blood–brain barrier and to reach brain cells if compared with the parental compound [54].

This evidence prompted us to investigate the therapeutic effect of GM1-OS in RTT murine models by analyzing its capability to rescue pathological phenotypes both in vitro and in vivo.

First, we focused on the GM1-OS mechanism of action, demonstrating that GM1-OS, by working at the cell surface, is able to rescue the hypoactivation of PM Trk receptors (Figure 4). It can be noticed that the activation degree of the FL-TrkB receptor in vitro is higher (Figure 4b) if compared with the one obtained from cortices’ lysates (Figure 4b) upon GM1-OS treatment. It may be speculated that this difference arises from the presence of glial cells in the cortical tissue. In the nervous system, two main TrkB isoforms are expressed: the FL-tyrosine kinase receptor and the C-terminal truncated receptor TrkB.t1, lacking the catalytic tyrosine kinase domain [55]. At the cellular level, the FL-isoform is expressed in neurons, while TrkB.t1 is expressed both in neurons and in glial cells [56,57,58,59,60]. Through the expression of TrkB.t1 at the PM, non-neuronal cells control the amounts of extracellular BDNF by sequestering it in transport vesicles [55]. While no significant deregulation of truncated TrkB was observed in *Mecp2*-null cortices [61], it cannot be excluded that a specific altered expression in glial cells can affect the BDNF availability to neurons.

Coherently with its ability to bind and activate the TrkA/B receptors (Figure 4), which are known to activate a complex intracellular cascade playing a prominent role in the maintenance of synaptic structure and plasticity, GM1-OS induced a recovery of synaptic defects in *Mecp2*-ko neurons in vitro (Figure 1). Specifically, GM1-OS works by restoring the expression levels of the pre-synaptic Syn protein and the post-synaptic Shank2 protein, and especially their colocalization, which indicates a recovery of functional synapses, ultimately reinstating a physiological status.

Besides changes in protein expression, it is known that neuronal differentiation, maturation, and synaptogenesis are characterized by dramatic changes in lipid pattern. In particular, sialic acid-containing glycosphingolipids undergo strong variations during differentiation and synaptogenesis with the shift from simple (GM3, GD3) to complex ganglioside species (GM1, GD1a, GD1b, and GT1b) [42,62]. Accordingly, a deregulation of gangliosides’ pattern, in particular of GD1a and GT1b, has been reported in RTT [35,36,37]. Importantly, on PM, GD1a works as a reservoir of GM1 since the membrane-associated sialidase Neu3 (EC: 3.2.1.18) is able to produce GM1 from GD1a [63]. These modifications at the synaptic level are expected to alter the architecture of specific PM microdomains known as “lipid rafts,” thus influencing the synaptic function of RTT neurons [64]. In well accordance, patients carrying mutations in the gene codifying for GM3 synthase (EC: 2.4.3.9) have been reported to display RTT-like symptoms [65]. This enzyme is responsible for the generation of GM3, the biosynthetic precursor of all complex gangliosides [30,65]. Consistent with its capability to promote neuronal maturation, we have demonstrated that GM1-OS administration to primary granule neurons increases the expression of GM1 ganglioside levels in 5 h and decreases GM3 levels in 24 h [42]. Based on this premise, it would be interesting in future studies to address whether GM1-OS is able to stimulate the maturation of RTT neurons by modulating ganglioside metabolism.

Since RTT is also characterized by a harmful redox imbalance in which mitochondrial dysfunction plays a central role [21,25,26,27,28,29], we tested whether GM1-OS could impinge on mitochondrial mass and functioning. Previous data proposed an early deregulation of important nuclear and mitochondria encoded factors in MeCP2-deficient cells, resulting in an initial hyperpolarization of the mitochondria, which, in conjunction with changes in Oxphos complexes, determines a strong generation of ROS and ultimately compromises ATP production [25]. In our experimental conditions, no substantial reduction of mitochondrial mass or Oxphos complexes’ subunits was observed in *Mecp2*-null neurons, while GM1-OS treatment slightly influences the expression of complex IV and V (Figure 2). Although it is only a trend, this would be in accordance with previous observations demonstrating GM1-OS capability to boost mitochondria function by increasing mitochodria respiration, Oxphos complexes expression, and ATP production [44]. Interestingly, we verified that Mecp2 deficiency determines a mitochondrial O_2_^•−^ overload that is significantly reduced upon GM1-OS administration (Figure 2), confirming the antioxidant properties of GM1-OS observed in Parkinson’s [40,46] and Amyotrophic Lateral Sclerosis neuronal models [48].

Further, these in vitro findings on the trophic and protective activity of GM1-OS were validated in vivo using *Mecp2*-null mice. Specifically, we demonstrated that a 4-week treatment with GM1-OS significantly ameliorates almost all parameters assessed by Guy’s phenotypic score, indicating a general improvement of the well-being of RTT animals. The amelioration in motor performance was further verified by the accelerated rotarod test, reporting that the progressive impairment in motor abilities was countercacted by GM1-OS (Figure 3). These results are in line with GM1-OS capability to completely recover parkinsonism [41], a relevant aspect for RTT patients who show parkinsonian features [66]. Accordingly, RTT mice displayed a dopaminergic deficiency and motor impairment that were efficiently counteracted by treatment with L-Dopa [67,68].

By evaluating the survival of control and GM1-OS-treated WT and *Mecp2*-null mice after the end of treatment, no significant difference was observed among the experimental groups (Supplementary Figure S2), demonstrating the safety of GM1-OS treatment. In addition, this data could suggest that a lifelong GM1-OS administration would be required for maintaining the beneficial effects of the drug, and it would be in agreement with the evidence that GM1-OS is not internalized by cells (Figure 4) [38,42]. Moreover, since a halt in the symptomatology has been reported without a complete return to a healthy condition, we hypothesize that a treatment starting during early postnatal age could be more effective.

In line with this work, a recent study demonstrated the curative effect of NGF on the maturation and neuronal activity of cultured *Mecp2*-null neurons, as well as its in vivo efficacy in improving behavioral impairments in *Mecp2*-null mice [21]. Interestingly, NGF treatment induced a significant upregulation of Oxphos gene expression and the improvement of mitochondrial structure and respiration in the cortices of *Mecp2*-null mice. Additionally, an NGF-derived amelioration of neuronal maturation has been suggested by Gene Ontology analysis of RNA sequencing data. Given that GM1-OS acts as a Trk receptor agonist, potentiating the effects of neurotrophins, we would expect that GM1-OS may contribute to the preservation of synaptic activity and mitochondrial homeostasis in vivo by enhancing NTP signaling.

Collectively, our results demonstrate that GM1-OS is not internalized by cortical neurons, but it interacts with their PM. Here, it activates the Trk receptor, inducing a neurotrophic and neuroprotective program that stimulates the neuronal maturation as well as the maintenance of proper mitochondrial redox state, resulting in the amelioration of RTT-like symptoms in mice. Although we acknowledge that the molecular mechanisms and intracellular events modulated by GM1-OS are yet to be investigated in detail in this model, the data presented in this work demonstrate that GM1-OS could be an interesting drug candidate for RTT.

## 4. Materials and Methods

### 4.1. Materials

Commercial chemicals were of the highest purity available; common solvents were distilled before use, and water was doubly distilled in a glass apparatus.

Phosphate buffered saline (PBS), Calcium Magnesium Free (CMF)-PBS, glucose, paraformaldehyde (PFA), sucrose, sodium orthovanadate (Na_3_VO_4_), sodium chloride (NaCl), dithiothreitol (DTT), bovine serum albumin (BSA), phenylmethanesulfonyl fluoride (PMSF), protease inhibitor cocktail, aprotinin, Triton X-100, ethylenediaminetetraacetic acid (EDTA), sodium dodecyl sulfate (SDS), glycine, poly-D-lysine, methanol, triethylamine, chloroform, propanol, glycerol, Trizma base, blue bromophenol were from Sigma-Aldrich (St. Louis, MO, USA). L-Glutamine and penicillin/streptomycin solution were from EuroClone (Paignton, UK). MitoSOX™ Red superoxide indicator, Hoechst solution, Neurobasal medium, B27 Supplement, 4′,5-Diamidina-2-phenylindole (DAPI), Trypsin/EDTA, Dulbecco’s Modified Eagle Medium (DMEM), cell culture plates, FBS, Fluoromount- G™ Mounting Medium, and Hanks’ balanced salt solution (HBSS) were from Thermo Fisher Scientific (Waltham, MA, USA). An amount of 4–20% Mini-PROTEAN^®^ TGX™ Precast Protein Gels, Turbo Polyvinylidene Difluoride (PVDF) Mini-Midi membrane, and DC™ protein assay kit were from BioRad (Hercules, CA, USA). Poly-D-lysine-coated glass coverslips were from Neuvitro (Vancouver, WA, USA). Chemiluminescent kit for WB was from Cyanagen (Bologna, Italy). Ultima gold was from Perkin Elmer (Waltham, MA, USA). High performance thin layer chromatography (HPTLC) plates were from Merk Millipore (Burlington, MA, USA).

### 4.2. Antibodies

For immunofluorescence analyses on neurons, the following antibodies were used: chicken polyclonal anti-Syn (research resource identifier, RRID:AB_2622240), mouse monoclonal anti-Shank2 (RRID:AB_2661874) purchased from Synaptic System (Göttingen, Germany), and polyclonal anti-Map2 (RRID:AB_2722660) from Cell Signaling Technology (Danvers, MA, USA). Secondary anti-chicken, anti-mouse, and anti-rabbit antibodies (Alexa Fluor 488, 547, and 633, respectively) were purchased from Thermo Fisher Scientific (Waltham, MA, USA).

For WB analyses, the following antibodies were used: rabbit polyclonal anti-TOM20 (RRID: AB_2207530) purchased from ProteinTech (Manchester, UK); rabbit monoclonal anti-HTRA2 (RRID: AB_11220423), rabbit polyclonal anti-TrkA (RRID: AB_10695253), mouse monoclonal anti-β-actin (RRID:AB_2242334), rabbit monoclonal anti-TrkB (RRID:AB_2155125), rabbit monoclonal anti-phospho-TrkA^Tyr674/675^/TrkB^Tyr706/707^ (RRID:AB_916186), and secondary horseradish peroxidase (HRP)-conjugated anti-rabbit IgG (RRID: AB_2099233) from Cell Signaling Technology (Danvers, MA, USA); mouse Oxphos cocktail (RRID: AB_2629281) from Abcam (Cambridge, UK); rabbit polyclonal anti-phospho-TrkB^Tyr816^ and rabbit polyclonal anti-GADPH (RRID: AB_796208) were from Merck Millipore (Burlington, MA, USA); mouse monoclonal anti-calnexin antibody (RRID: AB 397883) from BD Biosciences (San Jose, CA, USA); secondary HRP-conjugated goat anti-mouse IgG (H + L) antibody (RRID: AB_228309) from Life Technologies (Carlsbad, CA, USA); anti-HSP90 (RRID:AB_675659, and mouse monoclonal anti-phospho-TrkA^Tyr490^ (RRID: 628399) antibody from Santa Cruz (Dallas, TX, USA).

### 4.3. Animals

All animal procedures were executed in accordance with the European Union Communities Council Directive (2010/63/EU) and Italian laws (D.L.26/2014). Protocols were approved by the Italian Council on Animal Care in accordance with the Italian law (Italian Government decree No. 210/2017 PR).

Animals were housed with a 12 h light/12 h dark cycle in a temperature- and humidity-controlled environment with ad libitum access to food and water.

The experiments were performed on the *Mecp2*^tm1.1Bird^ mouse model in the outbred CD1 genetic background that recapitulates the typical RTT phenotype of C57BL/6 mice and, at the same time, is characterized by a large progeny and better maternal care [6]. Mice with the different genotypes were obtained by crossing *Mecp2* heterozygous females with WT male mice; the latter were purchased from Charles River Laboratories.

Mouse genotype was determined by polymerase chain reaction (PCR) using the following primers: 5′-ACCTAGCCTGCCTGTACTTT-3′ forward primer for null allele; 5′ GACTGAAGTTACAGATGGTTGTG- 3′ forward primer for WT type allele; 5′ CCACCCTCCAGTTTGGTTTA-3′ as common reverse primer [6].

### 4.4. Animals’ Treatment

GM1-OS was dissolved in saline physiological solution at a concentration of 4 mg/mL and was IP injected at 20 mg/kg body weight daily for 4 weeks.

*Mecp2*-ko (P35–37) and WT at the corresponding age were randomly assigned to the experimental groups (WT + saline, *n* = 11; *Mecp2*-ko + saline, *n* = 10; *Mecp2*-ko + GM1-OS, *n* = 9). Two hours before sacrifice, mice received the last injection of saline/GM1-OS. Then, animals were euthanized by cervical dislocation and rapid decapitation, and cerebral cortices were isolated.

The dose, the administration route, and the treatment duration were chosen based on the recovery of parkinsonism in *B4galnt1*^+/−^ mice [41].

### 4.5. Behavioral Analyses

All the animals were handled for five days before starting behavioral experiments. After an acclimatization period of 30 min, each experiment was conducted in a separate behavioral testing room during the light cycle. The animal’s experimental condition was unknown to the researcher during the execution of tests.

#### 4.5.1. Phenotypic Characterization

Using a previously reported scoring method, the presence or absence of RTT-like symptoms was assessed in *Mecp2*-ko and WT littermates [6,52]. An observer, blind to the treatment, assigned a score to general condition, mobility, gait, hindlimb clasping, and tremor at each session. Mouse symptoms were scored according to the following scale: 0 if the symptom was absent, 1 if the symptom was present but mild, and 2 when the symptom was severe. Mice were allowed to freely move on the bench in order to test their mobility, and the movement’s spontaneity was assessed. Breathing was evaluated by observing the movement of flanks when animal is still, distinguishing between normal breathing, breathing with pauses, or rapid breathing until gasping/panting. Hindlimb clasping was measured by holding mice by the tail, while tremor was evaluated by placing mice on the palm hand. The evaluation of the fur integrity, eye crustiness, and posture are included in the expression “general conditions”. Notably, due to ethical concerns, every animal that either lost weight quickly or scored 2 on the tremor assessment was euthanized. Phenotypic score evaluation was performed roughly two times per week. The progression of the symptomatology was graphically represented for each parameter by a cumulative plot (Figure 3), which was created by charting the score for each day that summed to the one of the days before it.

#### 4.5.2. Rotarod Test

Rotarod test was set up utilizing a five-lane Rotarod (Biological Research Apparatus, Ugo Basile, Varese, Italy) for mice. Animals were placed on the spinning rod (4 rpm) for 10 s to allow habituation before each experiment. During the test, the rotation speed was gradually increased from 4 rpm to 40 rpm every 30 s for a total of 300 s. Each mouse performed three trials every day that terminated when animal fell off the rod or achieved the maximum time (300 s) [53]. Rotarod test was executed before starting the treatment, 15 days and 30 days after saline/GM1-OS injection.

### 4.6. Primary Neuronal Cultures

For primary neuronal cultures, heterozygous females and WT mice were crossed to produce WT and *Mecp2* mutant embryos. Embryonic (E) day 0.5 was defined as the day of the vaginal plug, while E15.5 embryos were used to prepare primary neurons. Each embryo was genotyped by PCR as described above. Mice were sacrificed by cervical decapitation, and brains were removed under a microscope and immersed in ice-cold HBSS. Meninges were gently removed; cerebral cortex was rapidly dissected and maintained in ice-cold HBSS. Cortices were incubated with 0.25% Trypsin/EDTA for 7 min at 37 °C. Trypsin was eliminated by washing with HBSS and inactivated in DMEM supplemented with 10% FBS and 1% penicillin/streptomycin. By gentle pipetting, cortices were then mechanically dissociated in DMEM containing 10% FBS, 1% L-Glutamine, 1% penicillin/streptomycin. Neurons were seeded in Neurobasal medium supplemented with 2% B27, 1% L-Glutamine and 1% penicillin/streptomycin on poly-D-lysine-coated glass coverslips in 24-well plates (40,000 cells/well) for immunofluorescence analyses or in poly-D-lysine-coated (0.1 mg/mL) 6-well plates (250,000 cells/well) for WB experiments.

### 4.7. Cortical Neuron Treatments

GM1-OS was dissolved in water at a stock concentration of 2 mM, further diluted in the culture medium at the final concentration of 50 μM immediately after neurons’ plating or as specified below in the various experimental sections. In neuroblastoma and primary neuron cultures (including granule, dopaminergic, and motor neurons), concentrations of GM1-OS in the range of 50–100 µM have been consistently effective in inducing neuritogenic, neuroprotective, and neurorestorative effects through the activation of TrkA signaling [38,39,40,42,45,46,48]. Based on this evidence, in this study we selected the concentration of 50 µM to assess morphological and functional recovery in RTT neurons.

### 4.8. Fate of GM1-OS

The fate of GM1-OS administered to cortical neurons was verified using GM1-OS labeled with tritium on the external galactose, as previously described in literature [38,42]. Briefly, neurons were cultured in presence of 50 μM [^3^H]GM1-OS (45 μCi; specific radioactivity: 0.32 Ci/mmol) for 20 h. At the end of treatment, the medium was collected, and neurons were rinsed 3 times with culture medium containing 10% serum to collect the fraction of tritiated oligosaccharide that was weakly associated to the cell surface (SL fraction). Then, the cells were incubated with 0.1% trypsin solution for 1 min to obtain the portion that was strongly linked to the cell surface (TL fraction). Finally, cells were lysed for collecting the fraction of radiolabeled GM1-OS being internalized by the neurons (TS fraction). Using liquid scintillation counting, the radioactivity of each fraction and the collected medium was analyzed.

### 4.9. Immunofluorescence and Analysis of Synaptic Puncta

Cortical neurons (DIV14), seeded on glass coverslips and treated with vehicle or GM1-OS for 14 days, were fixed for 8 min with 4% PFA dissolved in PBS with 10% sucrose at 23 °C and washed three times with PBS for 1 min. Cells were permeabilized in PBS containing 0.2% Triton X-100 for 3 min on ice, washed with 0.2% BSA in PBS, and then blocked with 4% BSA in PBS for 15 min. Then, cells were incubated with primary antibody for 16 h (4 °C). Primary antibodies were diluted in 0.2% BSA in PBS as follows: anti-Syn (1:500), anti-Shank2 (1:300), and anti-Map2 (1:1000). After washing in BSA 0.2% in PBS, cells were incubated with the specific Alexa Fluor secondary antibody (1:500) in 0.2% BSA in PBS for 1 h at 23 °C. Finally, nuclei were stained with DAPI solution (1:1000 in PBS) for 10 min, and glass coverslips were mounted with Fluoromount-G™ Mounting Medium (Thermo Fisher Scientific, Waltham, MA, USA).

For the analysis of synaptic puncta, 144.88 × 144.88 µm^2^ Z-stack images (1024 × 1024 pixel resolution, 8-bit gray-scale depth) were acquired at 1× digital zoom using a 63× oil-immersion objective by laser-scanning confocal microscope (Zeiss LSM 800, Carl Zeiss, Boston, MA, USA) with a step size of 0.3 μm. For each dataset acquisition, parameters (offset background, digital gain, and laser intensity) were maintained constant among different experiments. By ImageJ software (2.14.0; Java 1.8.0_322, NIH, Bethesda, MD, USA; http://rsbweb.nih.gov/ij/), developed at the US National Institutes of Health [69], maximum intensity projection images were converted to binary images and processed with a fixed threshold for each channel acquired. The puncta density was calculated by counting only puncta lying along manually selected regions of interest (ROIs) within 20 μm of 3 primary branches/neuron. Only puncta with a minimum size of 0.16 μm^2^ were counted using Analyze Particles Plugin of Image J (2.14.0; Java 1.8.0_322, NIH, Bethesda, USA; http://rsbweb.nih.gov/ij/).

For each collected Z-stack image, the ImageJ Plugin Colocalization highlighter(2.14.0; Java 1.8.0_322, NIH, Bethesda, MD, USA; http://rsbweb.nih.gov/ij/) was used to evaluate the puncta co-localization of pre- and post-synaptic markers. In manually chosen ROIs of the binary mask derived from the maximum intensity projection, co-localized puncta were measured. Only puncta with a minimum size of 0.1 μm^2^ were counted [49,50].

### 4.10. MitoSOX Red Staining

Cortical neurons (DIV7), treated with vehicle or GM1-OS for 7 days, were incubated with 2 μM MitoSOX™ Red reagent (Thermo Fisher Scientific, Waltham, MA, USA) in HBSS with Ca^2+^ and Mg^2+^ for 10 min at 37 °C. At the end of the incubation, the cells were rinsed in PBS and fixed in 4% PFA in PBS for 8 min at 23 °C. Nuclei staining was performed by Hoechst dye (1:500 in PBS, 5 min, 23 °C), and slides were mounted with Fluoromount-G™ reagent (Thermo Fisher Scientific, Waltham, MA, USA). Images were acquired using a Nikon Eclipse Ni upright microscope (Nikon, Amstelveen, The Netherlands) with a 40× objective, and, for each experiment, acquiring conditions were maintained identical between the different experimental conditions. At least 20 fields for each condition were acquired for each experiment. Red signal was quantified with ImageJ (2.14.0; Java 1.8.0_322, NIH, Bethesda, MD, USA; http://rsbweb.nih.gov/ij/) by setting a fixed threshold and dividing the area value per the total number of nuclei.

### 4.11. WB Analysis

Cortical neurons incubated with vehicle or GM1-OS for 7 days were washed twice with cold PBS containing Na_3_VO_4_ (1 mM), lysed with 30 μL/well for 6-well plates of Laemmli buffer (0.15 M DTT, 94 mM Tris-HCl, 15% glycerol, *v*/*v*, 3% SDS *w*/*v*, 0.015% blue bromophenol, *v*/*v*), and sonicated with Vibra-cell^TM^ (Sonics & Materials, Inc., Danbury, CT, USA) for 3 times (10 s at 30% amplitude) to analyze mitochondrial proteins. To study the Trk activation, neurons were cultured for 7 days and treated at DIV7 for 10 min with GM1-OS/vehicle before being collected and treated as described above.

Cortices were lysed in ice-cold lysis buffer (50 mM Tris–HCl, pH 7.4, 150 mM NaCl, 5 mM EDTA, 1% Triton X-100, 1 mM Na_3_VO_4_, 1 mM PMSF, 2% (*v*/*v*) aprotinin, and 1% (*v*/*v*) protease inhibitor cocktail) using a potter pestle, then left for 30 min on ice and sonicated for 10 s at 30 amplitude on ice with Vibra-cell^TM^ (Sonics & Materials, Inc. Danbury, CT, USA). Lysates were clarified by centrifugation for 30 min at 10,000 g (4 °C). Protein concentrations were evaluated by DC™ protein assay kit (Bio-Rad, Hercules, CA, USA). Protein lysates were diluted in Laemmli buffer and boiled at 99 °C for 5 min.

Equal amounts of proteins were separated on a 4–20% precast or 8% polyacrylamide gel and transferred to PVDF membranes using the Trans-Blot^®^ Turbo^TM^ Transfer System (Bio-Rad, Hercules, CA, USA). For correct Oxphos subunit detection, lysate boiling was avoided. PVDF membranes were incubated for 1 h in blocking solution [Tris-buffered saline containing 0.1% Tween-20 (TBS-T) and 5% non-fat dry milk] and then incubated 16 h (4 °C) with primary antibodies as follows: anti-TOM20 (1:1000 in 5% milk in TBS-T), anti-HTRA2 (1:1000 in 5% milk in TBS-T), anti-Oxphos subunits (1:1000 in 3% BSA in TBS-T), anti-HSP90 (1:1000 in 5% milk in TBS-T), anti-calnexin (1:2000 in 5% BSA in TBS-T), anti-TrkA (1:1000 in 5% BSA in TBS-T), anti-TrkB (1:1000 in 5% BSA in TBS-T), anti-phospho-TrkB^Tyr706/707^ (1:1000 in 5% BSA in TBS-T), anti-phospho-TrkB^Tyr816^ (1:500 in 5% BSA in TBS-T), anti-phospho-TrkA^Tyr490^ (1:500 in 5% milk in TBS-T), anti-β-actin (1:20,000 in 5% milk in TBS-T), anti-GAPDH (1:7000 in 5% BSA in TBS-T). After three washes in TBS-T, blots were incubated for 1 h at 23 °C with the appropriate HRP-conjugated secondary antibody (1:2000 in 5% milk in TBS-T or 5% BSA in TBS-T), and the immunocomplexes were visualized by using the enhanced luminol-based chemiluminescent substrate and the Uvitec system (Cleaver Scientific Ltd., Rugby, UK). Band quantification was performed using the ImageJ and data expressed as a percentage of WT controls (mean value of WT controls set to 100).

### 4.12. GM1-OS Preparation

The GM1-OS or [^3^H]GM1-OS was prepared by ozonolysis of GM1 or [^3^H]GM1 followed by alkaline degradation as previously reported [42,43,44]. To summarize, GM1 or [^3^H]GM1 was dissolved in methanol and subsequently progressively saturated with ozone at 23 °C. Following vacuum-assisted methanol evaporation, triethylamine was immediately added to the residue to bring the pH between 10.5 and 11.0. After 3 days, the triethylamine was evaporated, and GM1-oligosaccharides were purified by flash chromatography with chloroform–methanol–2-propanol–water (60:35:5:5 *v*–*v*–*v*–*v*) as eluent. The oligosaccharides were dissolved in methanol and stored at 4 °C. By nuclear magnetic resonance, mass spectrometry, and high-performance thin layer chromatography analyses, the homogeneity of GM1-OS or [^3^H]GM1-OS was verified to be over 99% [41].

### 4.13. Statistical Analysis

All values are expressed as mean ± SEM. Statistical analysis was performed by one-way or two-way ANOVA, followed by a Tukey’s multiple comparisons test or Fisher’s LSD post hoc tests. Additionally, Brown-Forsythe and Welch one-way ANOVA was employed. *p* < 0.05 was considered significant. The analysis was performed with Prism software 10.3.0 (GraphPad Software, Inc., La Jolla, CA, USA; https://www.graphpad.com/).

## Data Availability

The data presented in this study are available upon reasonable request to the corresponding authors.

## Supplementary Materials

The following supporting information can be downloaded at: https://www.mdpi.com/article/10.3390/ijms252111555/s1. “The graphical abstract was created with the BioRender softwar (https://app.biorender.com/) under the license citation number” Fazzari, M. (2024) BioRender.com/x59f275, accessed on 25 September 2024.

## Abbreviations

Ganglioside nomenclature is in accordance with IUPAC-IUBB recommendations [70]	
ATP	adenosine triphosphate
BBB	blood-brain-barrier
BDNF	brain-derived neurotrophic factor
BSA	bovine serum albumin
CMF-PBS	calcium magnesium free pbs
CTRL	control
DAPI	4′,5-diamina-2-phenylindole
DIV	day in vitro
DMEM	dulbecco’s modified eagles’ medium
DTT	dithiothreitol
E	embryonic day
EDTA	ethylenediaminetetraacetic acid
FBS	fetal bovine serum
FL-TrkB	full length-TrkB
GAPDH	glyceraldehyde-3-phosphate dehydrogenase
GDNF	glia cell-derived neurotrophic factor
GM1	II^3^Neu5Ac-GG_4_Cer, β-Gal-(1-3)-β-GalNac-(1-4)-[α-Neu5Ac-(2-3)]-β-Gal-(1-4)-β-Glc-Cer
GM1-OS	GM1-oligosaccharide, II^3^Neu5Ac-GG_4_, β-Gal-(1-3)-β-GalNac-(1-4)-[α-Neu5Ac-(2-3)]-β-Gal-(1-4)-Glc
HTRA2	High-temperature requirement protein A2
HBSS	hanks’ balanced salt solution
HPR	horseradish peroxidase
HSP90	heat shock protein 90
IP	intraperitoneal
KO	knockout
MAP2	microtubule associated protein 2
MeCP2	methyl-cpg binding protein 2 (human protein)
*MECP2*	methyl-cpg binding protein 2 (human gene)
Mecp2	methyl-cpg binding protein 2 (murine protein)
*Mecp2*	methyl-cpg binding protein 2 (murine gene)
MEK	mitogen-activated protein kinase kinase
MPTP	1-methyl-4-phenyl-1,2,3,6-tetrahydropyridine hydrocholoride
N2a	neuroblastoma Neuro2a
NaCl	sodium chloride
Na_3_VO_4_	sodium orthovanadate
NGF	nerve growth factor
NTP	neurotrophin
O_2_^•−^	superoxide anion
Oxphos	oxidative phosphorylation
P35–37	postnatal day 35–37
PBS	phosphate-buffered saline
PCR	polymerase chain reaction
PFA	paraformaldehyde
PM	plasma membrane
PMSF	phenylmethanesulfonyl fluoride
PVDF	polyvinylidene difluoride
ROI	regions of interest
ROS	reactive oxygen species
RRID	research resource identifier
RTT	Rett syndrome
SDS	sodium dodecyl sulfate
SEM	standard error of the mean
Shank2	SH3 and multiple ankyrin repeat domains protein 2
Syn	synapsin 1/2
SL	serum labile
TBS-T	tris-buffered saline containing 0.1% tween-20
TL	trypsin labile
TOM20	translocase of outer mitochondrial membrane 20
Trk	tropomyosin receptor kinase
TrkB.t1	C-terminal truncated receptor TrkB
TS	trypsin stabile
WB	western blotting
WT	wild-type
