# Supplementary material for: GM1 Oligosaccharide Ameliorates Rett Syndrome Phenotypes In Vitro and In Vivo via Trk Receptor Activation
Source: Int J Mol Sci. 2024 Oct 28;25(21):11555. doi: 10.3390/ijms252111555 (PMC11547101; doi:10.3390/ijms252111555)
Supplement: Supplementary file 1 [file ijms-25-11555-s001.zip › ijms-3255071-supplementary.pdf]

International Journal of
Molecular Sciences

MDPI

Supplementary information

GM1 oligosaccharide ameliorates Rett syndrome phenotypes in vitro and in vivo via Trk receptor activation

Maria Fazzari[1]*, Giulia Lunghi[1], Emma Veronica Carsana[1], Manuela Valsecchi[1], Eleonora Spiombi[1], Martina Breccia[1], Silvia Rosanna Casati[1], Silvia Pedretti[2], Nico Mitro[2,3], Laura Mauri[1], Maria Grazia Ciampa[1], Sandro Sonnino[1], Nicoletta Landsberger[1], Angelisa Frasca[1] and Elena Chiricozzi[1,]*

[1] Department of Medical Biotechnology and Translational Medicine. Università degli Studi di Milano, 20054 Segrate, Milan, Italy; giulia.lunghi@unimi.it (G.L.); emma.carsana@unimi.it (E.V.C); manuela.valsecchi@unimi.it (M.V.); eleonora.spiombi@gmail.com (E.S.); martina.breccia@unimi.it (M.B.); silvia.casati@unimi.it (S.R.C.); laura.mauri@unimi.it (L.M.); maria.ciampa@unimi.it (M.G.C.); sandro.sonnino@unimi.it (S.S.); nicoletta.landsberger@unimi.it (N.L.); angelisa.frasca@unimi.it (A.F.)
[2] Department of Pharmacological and Biomolecular Sciences "Rodolfo Paoletti". Università degli Studi di Milano, 20133 Milan, Italy; silvia.pedretti@unimi.it (S.P.); nico.mitro@unimi.it (N.M.)
[3] Department of Experimental Oncology, IEO, European Institute of Oncology IRCCS, 20139 Milan, Italy
* Correspondence: maria.fazzari@unimi.it (M.F.); elena.chiricozzi@unimi.it (E.C.)

1. Supplementary Figures

1.1. Supplementary Figure S1

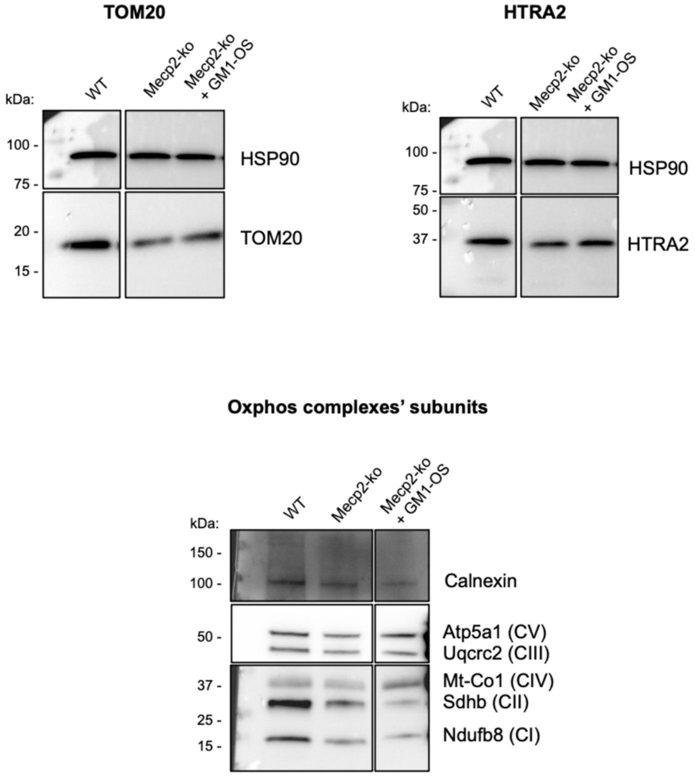

Figure S1. Mitochondrial proteins in WT and *Mecp2*-ko neurons. Representative images of WB analysis of mitochondrial proteins (TOM20, HTRA2 and Oxphos complexes subunits) performed on lysates from cortical neurons cultured in presence of 50 μM GM1-OS or vehicle for 7 days (DIV7). Mitochondrial protein bands were normalized to HSP90 and calnexin, used as loading controls.

1.2. Supplementary Figure S2

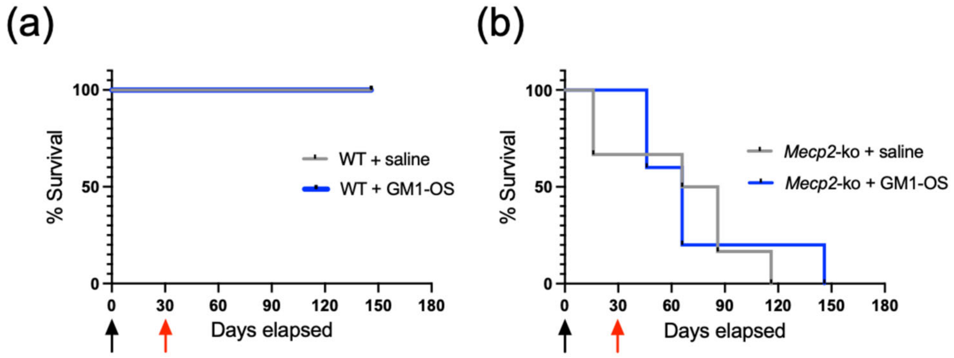

Figure S2. Survival analysis of WT and *Mecp2*-ko mice treated with saline or GM1-OS. WT and *Mecp2*-ko mice were treated with GM1-OS (20 mg/kg) or saline for 4 weeks, and health conditions and survival were monitored over time. Kaplan-Meyer survival curves show that GM1-OS treatment did not impact WT mice survival (**a**) and did not significantly extend lifespan in *Mecp2*-ko mice (**b**) compared to saline-receiving group. WT + saline, *n* = 6; WT + GM1-OS, *n* = 5; *Mecp2*-ko + saline, *n* = 6; *Mecp2*-ko + GM1-OS, *n* = 5; the survival curves were compared by Gehan-Breslow-Wilcoxon test. The black arrow marks the start of the treatment, and red arrow indicates the end of the treatment.

2. Supplementary Tables

Supplementary Table S1

	MOBILITY CUMULATIVE SCORE							
	**WT + saline**		**WT + GM1-OS**		***Mecp2*-ko + saline**		***Mecp2*-ko + GM1-OS**	
**Day**	Mean	SEM	Mean	SEM	Mean	SEM	Mean	SEM
**-1**	0.000	0.000	0.000	0.000	0.500	0.000	0.125	0.125
**+ 3**	0.000	0.000	0.000	0.000	0.900	0.058	0.625	0.214
**+ 7**	0.000	0.000	0.000	0.000	1.425	0.232	0.825	0.304
**+ 15**	0.000	0.000	0.000	0.000	2.200	0.274	1.425	0.272
**+ 18**	0.000	0.000	0.000	0.000	2.900	0.370	1.775	0.320
**+ 24**	0.000	0.000	0.000	0.000	3.600	0.478	2.125	0.368
**+ 28**	0.000	0.000	0.000	0.000	4.500	0.521	2.850	0.359

**Day - 1**	**Summary**	***p*-value**
WT + saline vs. *Mecp2*-ko + saline	ns	0.2683
WT + saline vs. *Mecp2*-ko + GM1-OS	ns	0.9683
WT + saline vs. WT + GM1-OS	ns	>0.9999
Mecp2-ko + saline vs. *Mecp2*-ko + GM1-OS	ns	0.5665
Mecp2-ko + saline vs. WT + GM1-OS	ns	0.2683
Mecp2-ko + GM1-OS vs. WT + GM1-OS	ns	0.9683

**Day + 3**	**Summary**	***p*-value**
WT + saline vs. *Mecp2*-ko + saline	**	0.0076
WT + saline vs. *Mecp2*-ko + GM1-OS	ns	0.1097
WT + saline vs. WT + GM1-OS	ns	> 0.9999
Mecp2-ko + saline vs. *Mecp2*-ko + GM1-OS	ns	0.7769
Mecp2-ko + saline vs. WT + GM1-OS	**	0.0076
Mecp2-ko + GM1-OS vs. WT + GM1-OS	ns	0.1097

**Day + 7**	**Summary**	***p*-value**
WT + saline vs. *Mecp2*-ko + saline	****	<0.0001
WT + saline vs. *Mecp2*-ko + GM1-OS	*	0.0172
WT + saline vs. WT + GM1-OS	ns	>0.9999
Mecp2-ko + saline vs. *Mecp2*-ko + GM1-OS	ns	0.168
Mecp2-ko + saline vs. WT + GM1-OS	****	<0.0001
Mecp2-ko + GM1-OS vs. WT + GM1-OS	*	0.0172

**Day + 15**	**Summary**	***p*-value**
WT + saline vs. *Mecp2*-ko + saline	****	<0.0001
WT + saline vs. *Mecp2*-ko + GM1-OS	****	<0.0001
WT + saline vs. WT + GM1-OS	ns	>0.9999
Mecp2-ko + saline vs. *Mecp2*-ko + GM1-OS	*	0.042
Mecp2-ko + saline vs. WT + GM1-OS	****	<0.0001
Mecp2-ko + GM1-OS vs. WT + GM1-OS	****	<0.0001

**Day + 18**	**Summary**	***p*-value**
WT + saline vs. *Mecp2*-ko + saline	****	<0.0001
WT + saline vs. *Mecp2*-ko + GM1-OS	****	<0.0001
WT + saline vs. WT + GM1-OS	ns	>0.9999
Mecp2-ko + saline vs. *Mecp2*-ko + GM1-OS	**	0.001
Mecp2-ko + saline vs. WT + GM1-OS	****	<0.0001
Mecp2-ko + GM1-OS vs. WT + GM1-OS	****	<0.0001

**Day + 24**	**Summary**	***p*-value**
WT + saline vs. *Mecp2*-ko + saline	****	<0.0001
WT + saline vs. *Mecp2*-ko + GM1-OS	****	<0.0001
WT + saline vs. WT + GM1-OS	ns	>0.9999
Mecp2-ko + saline vs. *Mecp2*-ko + GM1-OS	****	<0.0001
Mecp2-ko + saline vs. WT + GM1-OS	****	<0.0001
Mecp2-ko + GM1-OS vs. WT + GM1-OS	****	<0.0001

**Day + 28**	**Summary**	***p*-value**
WT + saline vs. *Mecp2*-ko + saline	****	<0.0001
WT + saline vs. *Mecp2*-ko + GM1-OS	****	<0.0001
WT + saline vs. WT + GM1-OS	ns	>0.9999
Mecp2-ko + saline vs. *Mecp2*-ko + GM1-OS	****	<0.0001
Mecp2-ko + saline vs. WT + GM1-OS	****	<0.0001
Mecp2-ko + GM1-OS vs. WT + GM1-OS	****	<0.0001

Supplementary Table S2

	BREATHING CUMULATIVE SCORE							
	**WT + saline**		**WT + GM1-OS**		***Mecp2*-ko + saline**		***Mecp2*-ko + GM1-OS**	
**Day**	Mean	SEM	Mean	SEM	Mean	SEM	Mean	SEM
**-1**	0.000	0.000	0.000	0.000	0.300	0.122	0.125	0.125
**+ 3**	0.000	0.000	0.000	0.000	0.300	0.122	0.275	0.189
**+ 7**	0.060	0.060	0.000	0.000	0.675	0.236	0.500	0.235
**+ 15**	0.060	0.060	0.000	0.000	1.525	0.263	1.150	0.260
**+ 18**	0.060	0.060	0.000	0.000	2.250	0.333	1.725	0.266
**+ 24**	0.060	0.060	0.000	0.000	2.975	0.405	2.300	0.292
**+ 28**	0.060	0.060	0.000	0.000	3.925	0.453	3.150	0.266

**Day - 1**	**Summary**	***p*-value**
WT + saline vs. *Mecp2*-ko + saline	ns	0.6042
WT + saline vs. *Mecp2*-ko + GM1-OS	ns	0.9551
WT + saline vs. WT + GM1-OS	ns	>0.9999
Mecp2-ko + saline vs. *Mecp2*-ko + GM1-OS	ns	0.9025
Mecp2-ko + saline vs. WT + GM1-OS	ns	0.6042
Mecp2-ko + GM1-OS vs. WT + GM1-OS	ns	0.9551

**Day + 3**	**Summary**	***p*-value**
WT + saline vs. *Mecp2*-ko + saline	ns	0.6042
WT + saline vs. *Mecp2*-ko + GM1-OS	ns	0.6688
WT + saline vs. WT + GM1-OS	ns	>0.9999
Mecp2-ko + saline vs. *Mecp2*-ko + GM1-OS	ns	0.9997
Mecp2-ko + saline vs. WT + GM1-OS	ns	0.6042
Mecp2-ko + GM1-OS vs. WT + GM1-OS	ns	0.6688

**Day + 7**	**Summary**	***p*-value**
WT + saline vs. *Mecp2*-ko + saline	ns	0.0602
WT + saline vs. *Mecp2*-ko + GM1-OS	ns	0.272
WT + saline vs. WT + GM1-OS	ns	0.9936
Mecp2-ko + saline vs. *Mecp2*-ko + GM1-OS	ns	0.9025
Mecp2-ko + saline vs. WT + GM1-OS	*	0.032
Mecp2-ko + GM1-OS vs. WT + GM1-OS	ns	0.1723

**Day + 15**	**Summary**	***p*-value**
WT + saline vs. *Mecp2*-ko + saline	****	<0.0001

WT + saline vs. *Mecp2*-ko + GM1-OS	***	0.0001
WT + saline vs. WT + GM1-OS	ns	0.9936
Mecp2-ko + saline vs. *Mecp2*-ko + GM1-OS	ns	0.4604
Mecp2-ko + saline vs. WT + GM1-OS	****	<0.0001
Mecp2-ko + GM1-OS vs. WT + GM1-OS	****	<0.0001

**Day + 18**	**Summary**	***p*-value**
WT + saline vs. *Mecp2*-ko + saline	****	<0.0001
WT + saline vs. *Mecp2*-ko + GM1-OS	****	<0.0001
WT + saline vs. WT + GM1-OS	ns	0.9936
Mecp2-ko + saline vs. *Mecp2*-ko + GM1-OS	ns	0.1751
Mecp2-ko + saline vs. WT + GM1-OS	****	<0.0001
Mecp2-ko + GM1-OS vs. WT + GM1-OS	****	<0.0001

**Day + 24**	**Summary**	***p*-value**
WT + saline vs. *Mecp2*-ko + saline	****	<0.0001
WT + saline vs. *Mecp2*-ko + GM1-OS	****	<0.0001
WT + saline vs. WT + GM1-OS	ns	0.9936
Mecp2-ko + saline vs. *Mecp2*-ko + GM1-OS	*	0.0464
Mecp2-ko + saline vs. WT + GM1-OS	****	<0.0001
Mecp2-ko + GM1-OS vs. WT + GM1-OS	****	<0.0001

**Day + 28**	**Summary**	***p*-value**
WT + saline vs. *Mecp2*-ko + saline	****	<0.0001
WT + saline vs. *Mecp2*-ko + GM1-OS	****	<0.0001
WT + saline vs. WT + GM1-OS	ns	0.9936
Mecp2-ko + saline vs. *Mecp2*-ko + GM1-OS	*	0.016
Mecp2-ko + saline vs. WT + GM1-OS	****	<0.0001
Mecp2-ko + GM1-OS vs. WT + GM1-OS	****	<0.0001

Supplementary Table S3

	GAIT CUMULATIVE SCORE							
	**WT + saline**		**WT + GM1-OS**		***Mecp2*-ko + saline**		***Mecp2*-ko + GM1-OS**	
**Day**	Mean	SEM	Mean	SEM	Mean	SEM	Mean	SEM
**-1**	0.000	0.000	0.000	0.000	0.250	0.144	0.250	0.144
**+ 3**	0.000	0.000	0.000	0.000	0.850	0.202	0.850	0.087
**+ 7**	0.060	0.060	0.000	0.000	1.500	0.255	1.300	0.071
**+ 15**	0.060	0.060	0.000	0.000	2.275	0.225	1.950	0.087
**+ 18**	0.060	0.060	0.000	0.000	2.925	0.189	2.525	0.125
**+ 24**	0.060	0.060	0.000	0.000	3.575	0.189	3.100	0.187
**+ 28**	0.060	0.060	0.000	0.000	4.475	0.155	3.925	0.250

**Day - 1**	**Summary**	***p*-value**
WT + saline vs. *Mecp2*-ko + saline	ns	0.3966
WT + saline vs. *Mecp2*-ko + GM1-OS	ns	0.3966
WT + saline vs. WT + GM1-OS	ns	>0.9999
Mecp2-ko + saline vs. *Mecp2*-ko + GM1-OS	ns	>0.9999
Mecp2-ko + saline vs. WT + GM1-OS	ns	0.3966
Mecp2-ko + GM1-OS vs. WT + GM1-OS	ns	0.3966

**Day + 3**	**Summary**	***p*-value**
WT + saline vs. *Mecp2*-ko + saline	****	<0.0001
WT + saline vs. *Mecp2*-ko + GM1-OS	****	<0.0001
WT + saline vs. WT + GM1-OS	ns	>0.9999
Mecp2-ko + saline vs. *Mecp2*-ko + GM1-OS	ns	>0.9999
Mecp2-ko + saline vs. WT + GM1-OS	****	<0.0001
Mecp2-ko + GM1-OS vs. WT + GM1-OS	****	<0.0001

**Day + 7**	**Summary**	***p*-value**
WT + saline vs. *Mecp2*-ko + saline	****	<0.0001
WT + saline vs. *Mecp2*-ko + GM1-OS	****	<0.0001
WT + saline vs. WT + GM1-OS	ns	0.978
Mecp2-ko + saline vs. *Mecp2*-ko + GM1-OS	ns	0.6304
Mecp2-ko + saline vs. WT + GM1-OS	****	<0.0001
Mecp2-ko + GM1-OS vs. WT + GM1-OS	****	<0.0001

**Day + 15**	**Summary**	***p*-value**
WT + saline vs. *Mecp2*-ko + saline	****	<0.0001

WT + saline vs. *Mecp2*-ko + GM1-OS	****	<0.0001
WT + saline vs. WT + GM1-OS	ns	0.978
Mecp2-ko + saline vs. *Mecp2*-ko + GM1-OS	ns	0.2165
Mecp2-ko + saline vs. WT + GM1-OS	****	<0.0001
Mecp2-ko + GM1-OS vs. WT + GM1-OS	****	<0.0001

**Day + 18**	**Summary**	***p*-value**
WT + saline vs. *Mecp2*-ko + saline	****	<0.0001
WT + saline vs. *Mecp2*-ko + GM1-OS	****	<0.0001
WT + saline vs. WT + GM1-OS	ns	0.978
Mecp2-ko + saline vs. *Mecp2*-ko + GM1-OS	ns	0.0851
Mecp2-ko + saline vs. WT + GM1-OS	****	<0.0001
Mecp2-ko + GM1-OS vs. WT + GM1-OS	****	<0.0001

**Day + 24**	**Summary**	***p*-value**
WT + saline vs. *Mecp2*-ko + saline	****	<0.0001
WT + saline vs. *Mecp2*-ko + GM1-OS	****	<0.0001
WT + saline vs. WT + GM1-OS	ns	0.978
Mecp2-ko + saline vs. *Mecp2*-ko + GM1-OS	*	0.0275
Mecp2-ko + saline vs. WT + GM1-OS	****	<0.0001
Mecp2-ko + GM1-OS vs. WT + GM1-OS	****	<0.0001

**Day + 28**	**Summary**	***p*-value**
WT + saline vs. *Mecp2*-ko + saline	****	<0.0001
WT + saline vs. *Mecp2*-ko + GM1-OS	****	<0.0001
WT + saline vs. WT + GM1-OS	ns	0.978
Mecp2-ko + saline vs. *Mecp2*-ko + GM1-OS	**	0.0075
Mecp2-ko + saline vs. WT + GM1-OS	****	<0.0001
Mecp2-ko + GM1-OS vs. WT + GM1-OS	****	<0.0001

Supplementary Table S4

	CLASPING CUMULATIVE SCORE							
	**WT + saline**		**WT + GM1-OS**		***Mecp2*-ko + saline**		***Mecp2*-ko + GM1-OS**	
**Day**	Mean	SEM	Mean	SEM	Mean	SEM	Mean	SEM
**-1**	0.000	0.000	0.000	0.000	0.275	0.103	0.325	0.118
**+ 3**	0.000	0.000	0.000	0.000	0.725	0.075	0.675	0.149
**+ 7**	0.000	0.000	0.000	0.000	1.400	0.091	1.275	0.103
**+ 15**	0.000	0.000	0.000	0.000	2.100	0.212	1.850	0.166
**+ 18**	0.000	0.000	0.000	0.000	2.825	0.266	2.500	0.235
**+ 24**	0.000	0.000	0.000	0.000	3.550	0.328	3.150	0.312
**+ 28**	0.000	0.000	0.000	0.000	4.500	0.339	3.875	0.347

**Day - 1**	**Summary**	***p*-value**
WT + saline vs. *Mecp2*-ko + saline	ns	0.5085
WT + saline vs. *Mecp2*-ko + GM1-OS	ns	0.3597
WT + saline vs. WT + GM1-OS	ns	>0.9999
Mecp2-ko + saline vs. *Mecp2*-ko + GM1-OS	ns	0.9951
Mecp2-ko + saline vs. WT + GM1-OS	ns	0.5085
Mecp2-ko + GM1-OS vs. WT + GM1-OS	ns	0.3597

**Day + 3**	**Summary**	***p*-value**
WT + saline vs. *Mecp2*-ko + saline	**	0.0022
WT + saline vs. *Mecp2*-ko + GM1-OS	**	0.0051
WT + saline vs. WT + GM1-OS	ns	>0.9999
Mecp2-ko + saline vs. *Mecp2*-ko + GM1-OS	ns	0.9951
Mecp2-ko + saline vs. WT + GM1-OS	**	0.0022
Mecp2-ko + GM1-OS vs. WT + GM1-OS	**	0.0051

**Day + 7**	**Summary**	***p*-value**
WT + saline vs. *Mecp2*-ko + saline	****	<0.0001
WT + saline vs. *Mecp2*-ko + GM1-OS	****	<0.0001
WT + saline vs. WT + GM1-OS	ns	>0.9999
Mecp2-ko + saline vs. *Mecp2*-ko + GM1-OS	ns	0.932
Mecp2-ko + saline vs. WT + GM1-OS	****	<0.0001
Mecp2-ko + GM1-OS vs. WT + GM1-OS	****	<0.0001

**Day + 15**	**Summary**	***p*-value**
WT + saline vs. *Mecp2*-ko + saline	****	<0.0001

WT + saline vs. *Mecp2*-ko + GM1-OS	****	<0.0001
WT + saline vs. WT + GM1-OS	ns	>0.9999
Mecp2-ko + saline vs. *Mecp2*-ko + GM1-OS	ns	0.629
Mecp2-ko + saline vs. WT + GM1-OS	****	<0.0001
Mecp2-ko + GM1-OS vs. WT + GM1-OS	****	<0.0001

**Day + 18**	**Summary**	***p*-value**
WT + saline vs. *Mecp2*-ko + saline	****	<0.0001
WT + saline vs. *Mecp2*-ko + GM1-OS	****	<0.0001
WT + saline vs. WT + GM1-OS	ns	>0.9999
Mecp2-ko + saline vs. *Mecp2*-ko + GM1-OS	ns	0.407
Mecp2-ko + saline vs. WT + GM1-OS	****	<0.0001
Mecp2-ko + GM1-OS vs. WT + GM1-OS	****	<0.0001

**Day + 24**	**Summary**	***p*-value**
WT + saline vs. *Mecp2*-ko + saline	****	<0.0001
WT + saline vs. *Mecp2*-ko + GM1-OS	****	<0.0001
WT + saline vs. WT + GM1-OS	ns	>0.9999
Mecp2-ko + saline vs. *Mecp2*-ko + GM1-OS	ns	0.2272
Mecp2-ko + saline vs. WT + GM1-OS	****	<0.0001
Mecp2-ko + GM1-OS vs. WT + GM1-OS	****	<0.0001

**Day + 28**	**Summary**	***p*-value**
WT + saline vs. *Mecp2*-ko + saline	****	<0.0001
WT + saline vs. *Mecp2*-ko + GM1-OS	****	<0.0001
WT + saline vs. WT + GM1-OS	ns	>0.9999
Mecp2-ko + saline vs. *Mecp2*-ko + GM1-OS	*	0.0179
Mecp2-ko + saline vs. WT + GM1-OS	****	<0.0001
Mecp2-ko + GM1-OS vs. WT + GM1-OS	****	<0.0001

Supplementary Table S5

	TREMOR CUMULATIVE SCORE							
	**WT + saline**		**WT + GM1-OS**		***Mecp2*-ko + saline**		***Mecp2*-ko + GM1-OS**	
**Day**	Mean	SEM	Mean	SEM	Mean	SEM	Mean	SEM
**-1**	0.000	0.000	0.000	0.000	0.550	0.050	0.400	0.058
**+ 3**	0.000	0.000	0.000	0.000	1.050	0.050	0.850	0.096
**+ 7**	0.100	0.063	0.000	0.000	1.625	0.075	1.225	0.138
**+ 15**	0.100	0.063	0.000	0.000	2.450	0.166	1.825	0.048
**+ 18**	0.100	0.063	0.000	0.000	3.300	0.178	2.475	0.048
**+ 24**	0.100	0.063	0.000	0.000	4.150	0.202	3.125	0.131
**+ 28**	0.100	0.063	0.000	0.000	5.200	0.235	3.900	0.082

**Day - 1**	**Summary**	***p*-value**
WT + saline vs. *Mecp2*-ko + saline	****	<0.0001
WT + saline vs. *Mecp2*-ko + GM1-OS	**	0.0062
WT + saline vs. WT + GM1-OS	ns	>0.9999
Mecp2-ko + saline vs. *Mecp2*-ko + GM1-OS	ns	0.6338
Mecp2-ko + saline vs. WT + GM1-OS	****	<0.0001
Mecp2-ko + GM1-OS vs. WT + GM1-OS	**	0.0062

**Day + 3**	**Summary**	***p*-value**
WT + saline vs. *Mecp2*-ko + saline	****	<0.0001
WT + saline vs. *Mecp2*-ko + GM1-OS	****	<0.0001
WT + saline vs. WT + GM1-OS	ns	>0.9999
Mecp2-ko + saline vs. *Mecp2*-ko + GM1-OS	ns	0.39
Mecp2-ko + saline vs. WT + GM1-OS	****	<0.0001
Mecp2-ko + GM1-OS vs. WT + GM1-OS	****	<0.0001

**Day + 7**	**Summary**	***p*-value**
WT + saline vs. *Mecp2*-ko + saline	****	<0.0001
WT + saline vs. *Mecp2*-ko + GM1-OS	****	<0.0001
WT + saline vs. WT + GM1-OS	ns	0.8111
Mecp2-ko + saline vs. *Mecp2*-ko + GM1-OS	*	0.0106
Mecp2-ko + saline vs. WT + GM1-OS	****	<0.0001
Mecp2-ko + GM1-OS vs. WT + GM1-OS	****	<0.0001

**Day + 15**	**Summary**	***p*-value**
WT + saline vs. *Mecp2*-ko + saline	****	<0.0001
WT + saline vs. *Mecp2*-ko + GM1-OS	****	<0.0001

WT + saline vs. WT + GM1-OS	ns	0.8111
Mecp2-ko + saline vs. *Mecp2*-ko + GM1-OS	****	<0.0001
Mecp2-ko + saline vs. WT + GM1-OS	****	<0.0001
Mecp2-ko + GM1-OS vs. WT + GM1-OS	****	<0.0001

**Day + 18**	**Summary**	***p*-value**
WT + saline vs. *Mecp2*-ko + saline	****	<0.0001
WT + saline vs. *Mecp2*-ko + GM1-OS	****	<0.0001
WT + saline vs. WT + GM1-OS	ns	0.8111
Mecp2-ko + saline vs. *Mecp2*-ko + GM1-OS	****	<0.0001
Mecp2-ko + saline vs. WT + GM1-OS	****	<0.0001
Mecp2-ko + GM1-OS vs. WT + GM1-OS	****	<0.0001

**Day + 24**	**Summary**	***p*-value**
WT + saline vs. *Mecp2*-ko + saline	****	<0.0001
WT + saline vs. *Mecp2*-ko + GM1-OS	****	<0.0001
WT + saline vs. WT + GM1-OS	ns	0.8111
Mecp2-ko + saline vs. *Mecp2*-ko + GM1-OS	****	<0.0001
Mecp2-ko + saline vs. WT + GM1-OS	****	<0.0001
Mecp2-ko + GM1-OS vs. WT + GM1-OS	****	<0.0001

**Day + 28**	**Summary**	***p*-value**
WT + saline vs. *Mecp2*-ko + saline	****	<0.0001
WT + saline vs. *Mecp2*-ko + GM1-OS	****	<0.0001
WT + saline vs. WT + GM1-OS	ns	0.8111
Mecp2-ko + saline vs. *Mecp2*-ko + GM1-OS	****	<0.0001
Mecp2-ko + saline vs. WT + GM1-OS	****	<0.0001
Mecp2-ko + GM1-OS vs. WT + GM1-OS	****	<0.0001

Supplementary Table S6

	**GENERAL CONDITIONS CUMULATIVE SCORE**							
	**WT + saline**		**WT + GM1-OS**		***Mecp2*-ko + saline**		***Mecp2*-ko + GM1-OS**	
**Day**	Mean	SEM	Mean	SEM	Mean	SEM	Mean	SEM
**-1**	0.000	0.000	0.000	0.000	0.375	0.125	0.125	0.125
**+ 3**	0.000	0.000	0.000	0.000	0.775	0.278	0.625	0.236
**+ 7**	0.000	0.000	0.000	0.000	1.100	0.434	1.125	0.253
**+ 15**	0.000	0.000	0.000	0.000	1.825	0.379	1.775	0.307
**+ 18**	0.000	0.000	0.000	0.000	2.475	0.338	2.550	0.380
**+ 24**	0.000	0.000	0.000	0.000	3.125	0.315	3.325	0.464
**+ 28**	0.000	0.000	0.000	0.000	3.925	0.315	4.100	0.555

**Day - 1**	**Summary**	***p*-value**
WT + saline vs. *Mecp2*-ko + saline	ns	0.597
WT + saline vs. *Mecp2*-ko + GM1-OS	ns	0.9755
WT + saline vs. WT + GM1-OS	ns	>0.9999
Mecp2-ko + saline vs. *Mecp2*-ko + GM1-OS	ns	0.8587
Mecp2-ko + saline vs. WT + GM1-OS	ns	0.597
Mecp2-ko + GM1-OS vs. WT + GM1-OS	ns	0.9755

**Day + 3**	**Summary**	***p*-value**
WT + saline vs. *Mecp2*-ko + saline	ns	0.054
WT + saline vs. *Mecp2*-ko + GM1-OS	ns	0.166
WT + saline vs. WT + GM1-OS	ns	>0.9999
Mecp2-ko + saline vs. *Mecp2*-ko + GM1-OS	ns	0.9646
Mecp2-ko + saline vs. WT + GM1-OS	ns	0.054
Mecp2-ko + GM1-OS vs. WT + GM1-OS	ns	0.166

**Day + 7**	**Summary**	***p*-value**
WT + saline vs. *Mecp2*-ko + saline	**	0.0022
WT + saline vs. *Mecp2*-ko + GM1-OS	**	0.0017
WT + saline vs. WT + GM1-OS	ns	>0.9999
Mecp2-ko + saline vs. *Mecp2*-ko + GM1-OS	ns	0.9998
Mecp2-ko + saline vs. WT + GM1-OS	**	0.0022
Mecp2-ko + GM1-OS vs. WT + GM1-OS	**	0.0017

**Day + 15**	**Summary**	***p*-value**
WT + saline vs. *Mecp2*-ko + saline	****	<0.0001

WT + saline vs. *Mecp2*-ko + GM1-OS	****	<0.0001
WT + saline vs. WT + GM1-OS	ns	>0.9999
Mecp2-ko + saline vs. *Mecp2*-ko + GM1-OS	ns	0.9986
Mecp2-ko + saline vs. WT + GM1-OS	****	<0.0001
Mecp2-ko + GM1-OS vs. WT + GM1-OS	****	<0.0001

**Day + 18**	**Summary**	***p*-value**
WT + saline vs. *Mecp2*-ko + saline	****	<0.0001
WT + saline vs. *Mecp2*-ko + GM1-OS	****	<0.0001
WT + saline vs. WT + GM1-OS	ns	>0.9999
Mecp2-ko + saline vs. *Mecp2*-ko + GM1-OS	ns	0.9953
Mecp2-ko + saline vs. WT + GM1-OS	****	<0.0001
Mecp2-ko + GM1-OS vs. WT + GM1-OS	****	<0.0001

**Day + 24**	**Summary**	***p*-value**
WT + saline vs. *Mecp2*-ko + saline	****	<0.0001
WT + saline vs. *Mecp2*-ko + GM1-OS	****	<0.0001
WT + saline vs. WT + GM1-OS	ns	>0.9999
Mecp2-ko + saline vs. *Mecp2*-ko + GM1-OS	ns	0.9214
Mecp2-ko + saline vs. WT + GM1-OS	****	<0.0001
Mecp2-ko + GM1-OS vs. WT + GM1-OS	****	<0.0001

**Day + 28**	**Summary**	***p*-value**
WT + saline vs. *Mecp2*-ko + saline	****	<0.0001
WT + saline vs. *Mecp2*-ko + GM1-OS	****	<0.0001
WT + saline vs. WT + GM1-OS	ns	>0.9999
Mecp2-ko + saline vs. *Mecp2*-ko + GM1-OS	ns	0.9455
Mecp2-ko + saline vs. WT + GM1-OS	****	<0.0001
Mecp2-ko + GM1-OS vs. WT + GM1-OS	****	<0.0001

Supplementary Table S7

	CUMULATIVE PHENOTYPIC SCORE							
	**WT + saline**		**WT + GM1-OS**		***Mecp2*-ko + saline**		***Mecp2*-ko + GM1-OS**	
**Day**	Mean	SEM	Mean	SEM	Mean	SEM	Mean	SEM
**-1**	0.000	0.000	0.000	0.000	2.250	0.272	1.350	0.421
**+ 3**	0.000	0.000	0.000	0.000	4.600	0.460	3.900	0.505
**+ 7**	0.220	0.174	0.000	0.000	7.725	1.137	6.250	0.544
**+ 15**	0.220	0.174	0.000	0.000	12.375	1.322	9.975	0.562
**+ 18**	0.220	0.174	0.000	0.000	16.675	1.397	13.550	0.421
**+ 24**	0.220	0.174	0.000	0.000	20.975	1.521	17.125	0.312
**+ 28**	0.220	0.174	0.000	0.000	26.525	1.542	21.800	0.406

**Day - 1**	**Summary**	***p*-value**
WT + saline vs. *Mecp2*-ko + saline	*	0.0307
WT + saline vs. *Mecp2*-ko + GM1-OS	ns	0.3393
WT + saline vs. WT + GM1-OS	ns	>0.9999
Mecp2-ko + saline vs. *Mecp2*-ko + GM1-OS	ns	0.7127
Mecp2-ko + saline vs. WT + GM1-OS	*	0.0307
Mecp2-ko + GM1-OS vs. WT + GM1-OS	ns	0.3393

**Day + 3**	**Summary**	***p*-value**
WT + saline vs. *Mecp2*-ko + saline	****	<0.0001
WT + saline vs. *Mecp2*-ko + GM1-OS	****	<0.0001
WT + saline vs. WT + GM1-OS	ns	>0.9999
Mecp2-ko + saline vs. *Mecp2*-ko + GM1-OS	ns	0.8416
Mecp2-ko + saline vs. WT + GM1-OS	****	<0.0001
Mecp2-ko + GM1-OS vs. WT + GM1-OS	****	<0.0001

**Day + 7**	**Summary**	***p*-value**
WT + saline vs. *Mecp2*-ko + saline	****	<0.0001
WT + saline vs. *Mecp2*-ko + GM1-OS	****	<0.0001
WT + saline vs. WT + GM1-OS	ns	0.9914
Mecp2-ko + saline vs. *Mecp2*-ko + GM1-OS	ns	0.3077
Mecp2-ko + saline vs. WT + GM1-OS	****	<0.0001
Mecp2-ko + GM1-OS vs. WT + GM1-OS	****	<0.0001

**Day + 15**	**Summary**	***p*-value**
WT + saline vs. *Mecp2*-ko + saline	****	<0.0001

WT + saline vs. *Mecp2*-ko + GM1-OS	****	<0.0001
WT + saline vs. WT + GM1-OS	ns	0.9914
Mecp2-ko + saline vs. *Mecp2*-ko + GM1-OS	*	0.0281
Mecp2-ko + saline vs. WT + GM1-OS	****	<0.0001
Mecp2-ko + GM1-OS vs. WT + GM1-OS	****	<0.0001

**Day + 18**	**Summary**	***p*-value**
WT + saline vs. *Mecp2*-ko + saline	****	<0.0001
WT + saline vs. *Mecp2*-ko + GM1-OS	****	<0.0001
WT + saline vs. WT + GM1-OS	ns	0.9914
Mecp2-ko + saline vs. *Mecp2*-ko + GM1-OS	**	0.0021
Mecp2-ko + saline vs. WT + GM1-OS	****	<0.0001
Mecp2-ko + GM1-OS vs. WT + GM1-OS	****	<0.0001

**Day + 24**	**Summary**	***p*-value**
WT + saline vs. *Mecp2*-ko + saline	****	<0.0001
WT + saline vs. *Mecp2*-ko + GM1-OS	****	<0.0001
WT + saline vs. WT + GM1-OS	ns	0.9914
Mecp2-ko + saline vs. *Mecp2*-ko + GM1-OS	****	<0.0001
Mecp2-ko + saline vs. WT + GM1-OS	****	<0.0001
Mecp2-ko + GM1-OS vs. WT + GM1-OS	****	<0.0001

**Day + 28**	**Summary**	***p*-value**
WT + saline vs. *Mecp2*-ko + saline	****	<0.0001
WT + saline vs. *Mecp2*-ko + GM1-OS	****	<0.0001
WT + saline vs. WT + GM1-OS	ns	0.9914
Mecp2-ko + saline vs. *Mecp2*-ko + GM1-OS	****	<0.0001
Mecp2-ko + saline vs. WT + GM1-OS	****	<0.0001
Mecp2-ko + GM1-OS vs. WT + GM1-OS	****	<0.0001

Supplementary Table S8

ACCELERATED ROTAROD								
	**WT + saline**		**WT + GM1-OS**		***Mecp2*-ko + saline**		***Mecp2*-ko + GM1-OS**	
**Day**	Mean	SEM	Mean	SEM	Mean	SEM	Mean	SEM
**-1**	282.058	7.726	291.093	4.638	231.463	14.322	230.667	16.217
**+ 15**	294.909	4.799	299.500	0.500	145.213	21.936	202.556	20.613
**+ 28**	271.873	11.819	293.500	5.390	112.300	24.349	182.589	19.883

**Day - 1**	**Summary**	***p*-value**
WT + saline vs. WT + GM1-OS	ns	0.9783
WT + saline vs. *Mecp2*-ko + saline	ns	0.0522
WT + saline vs. *Mecp2*-ko + GM1-OS	ns	0.0566
WT + GM1-OS vs. *Mecp2*-ko + saline	ns	0.0532
WT + GM1-OS vs. *Mecp2*-ko + GM1-OS	ns	0.0557
Mecp2-ko + saline vs. *Mecp2*-ko + GM1-OS	ns	>0.9999

**Day + 15**	**Summary**	***p*-value**
WT + saline vs. WT + GM1-OS	ns	0.997
WT + saline vs. *Mecp2*-ko + saline	****	<0.0001
WT + saline vs. *Mecp2*-ko + GM1-OS	****	<0.0001
WT + GM1-OS vs. *Mecp2*-ko + saline	****	<0.0001
WT + GM1-OS vs. *Mecp2*-ko + GM1-OS	***	0.0005
Mecp2-ko + saline vs. *Mecp2*-ko + GM1-OS	*	0.0461

**Day + 28**	**Summary**	***p*-value**
WT + saline vs. WT + GM1-OS	ns	0.7743
WT + saline vs. *Mecp2*-ko + saline	****	<0.0001
WT + saline vs. *Mecp2*-ko + GM1-OS	***	0.0001
WT + GM1-OS vs. *Mecp2*-ko + saline	****	<0.0001
WT + GM1-OS vs. *Mecp2*-ko + GM1-OS	****	<0.0001
Mecp2-ko + saline vs. *Mecp2*-ko + GM1-OS	**	0.0087

Supplementary Table S9

	WEIGHT							
	**WT + saline**		**WT + GM1-OS**		***Mecp2*-ko + saline**		***Mecp2*-ko + GM1-OS**	
**Day**	Mean	SEM	Mean	SEM	Mean	SEM	Mean	SEM
**-1**	26.733	1.301	26.167	1.286	24.033	1.684	22.060	1.384
**+ 6**	29.067	1.303	28.017	0.983	26.067	1.720	25.260	1.473
**+ 14**	31.483	1.180	28.800	0.545	27.950	2.731	27.220	1.440
**+ 20**	33.033	1.245	31.483	0.667	29.875	2.979	28.160	1.899
**+ 27**	32.283	1.305	30.833	0.822	30.475	3.130	28.525	2.165

**Day - 1**	**Summary**	***p*-value**
WT + saline vs. WT + GM1-OS	ns	0.9925
WT + saline vs. *Mecp2*-ko + saline	ns	0.5515
WT + saline vs. *Mecp2*-ko + GM1-OS	ns	0.1363
WT + GM1-OS vs. *Mecp2*-ko + saline	ns	0.7238
WT + GM1-OS vs. *Mecp2*-ko + GM1-OS	ns	0.2283
Mecp2-ko + saline vs. *Mecp2*-ko + GM1-OS	ns	0.7935

**Day + 6**	**Summary**	***p*-value**
WT + saline vs. WT + GM1-OS	ns	0.9555
WT + saline vs. *Mecp2*-ko + saline	ns	0.4605
WT + saline vs. *Mecp2*-ko + GM1-OS	ns	0.2911
WT + GM1-OS vs. *Mecp2*-ko + saline	ns	0.7753
WT + GM1-OS vs. *Mecp2*-ko + GM1-OS	ns	0.5736
Mecp2-ko + saline vs. *Mecp2*-ko + GM1-OS	ns	0.9817

**Day + 14**	**Summary**	***p*-value**
WT + saline vs. WT + GM1-OS	ns	0.5566
WT + saline vs. *Mecp2*-ko + saline	ns	0.4139
WT + saline vs. *Mecp2*-ko + GM1-OS	ns	0.1994
WT + GM1-OS vs. *Mecp2*-ko + saline	ns	0.9823
WT + GM1-OS vs. *Mecp2*-ko + GM1-OS	ns	0.8816
Mecp2-ko + saline vs. *Mecp2*-ko + GM1-OS	ns	0.9898

**Day + 20**	**Summary**	***p*-value**
WT + saline vs. WT + GM1-OS	ns	0.8726

WT + saline vs. *Mecp2*-ko + saline	ns	0.5131
WT + saline vs. *Mecp2*-ko + GM1-OS	ns	0.1117
WT + GM1-OS vs. *Mecp2*-ko + saline	ns	0.8951
WT + GM1-OS vs. *Mecp2*-ko + GM1-OS	ns	0.4116
Mecp2-ko + saline vs. *Mecp2*-ko + GM1-OS	ns	0.8878

**Day + 27**	**Summary**	***p*-value**
WT + saline vs. WT + GM1-OS	ns	0.8929
WT + saline vs. *Mecp2*-ko + saline	ns	0.8579
WT + saline vs. *Mecp2*-ko + GM1-OS	ns	0.3585
WT + GM1-OS vs. *Mecp2*-ko + saline	ns	0.9986
WT + GM1-OS vs. *Mecp2*-ko + GM1-OS	ns	0.7434
Mecp2-ko + saline vs. *Mecp2*-ko + GM1-OS	ns	0.8635
